# Impact of NGOs’ undercover videos on citizens’ emotions and pro-social behaviors

**DOI:** 10.1038/s41598-024-68335-5

**Published:** 2024-09-04

**Authors:** Romain Espinosa, Sylvie Borau, Nicolas Treich

**Affiliations:** 1 CIRED, CNRS, Nogent-sur-Marne, France; 2 Toulouse Business School, Toulouse, France; 3 University of Toulouse Capitole, Toulouse School of Economics, INRAE, IAST, Toulouse, France

**Keywords:** NGOs, Undercover videos, Public engagement, Activism, Emotions, Animal welfare, Psychology and behaviour, Environmental economics

## Abstract

**Abstract:**

Undercover videos have become a popular tool among NGOs to influence public opinion and generate engagement for the NGO’s cause. These videos are seen as a powerful and cost-effective way of bringing about social change, as they provide first-hand evidence and generate a strong emotional response among those who see them. In this paper, we empirically assess the impact of undercover videos on support for the cause. We in addition analyze whether the increased engagement among viewers is driven by the negative emotional reactions produced by the video. To do so, we design an online experiment that enables us to estimate both the total and emotion-mediated treatment effects on engagement by randomly exposing participants to an undercover video (of animal abuse) and randomly introducing a cooling-off period. Using a representative sample of the French population (N=3,310), we find that the video successfully increases actions in favor of animals (i.e., donations to NGOs and petitions), but we fail to prove that this effect is due to the presence of primary emotions induced by the video. Last, we investigate whether activists correctly anticipate their undercover videos’ (emotional) impact via a prediction study involving activists (exploratory analysis).

**Protocol registration:**

This manuscript is a Stage-2 working paper of a Registered Report that received In-Principle-Acceptance from Scientific Reports on November 20th, 2023 [Link to Stage-1]. The Stage-1 that received In-Principal-Acceptance can be found here: https://osf.io/8cg2d.

## Introduction

NGOs aim to transform society by influencing the decisions of government officials, private businesses, and individuals^1–5^. However, activists typically face three major constraints in their attempts to challenge the status quo. The first is a resource constraint, as NGOs have significantly fewer resources to influence public opinion than private companies and governments. Second, NGOs face a credibility constraint, as individuals may assume that activists overstate their evidence in order to support their agenda. Last, there is an attention constraint, with NGOs often trying to raise awareness among people who are bombarded with information and for whom the issue at stake might seem more distant than other more-immediate matters.

In recent decades, technological advances have allowed activists to overcome these three constraints by directly providing evidence to the public. The development of small cameras and smartphones has facilitated the direct recording of evidence on sites to secretly gather information (i.e., undercover videos). The use of undercover videos has become a very cost-effective way of presenting unfiltered evidence to the public, and has loosened these resource constraints. Those who see these videos now have access to first-hand evidence that likely mitigates the credibility constraint, as this information is less likely to be distorted. Last, these videos often provoke strong emotional reactions among viewers, which captures their attention and generates engagement and virality on social media.

One of the objectives of undercover videos is to generate immediate public engagement for a cause, and most NGOs encourage individuals to sign a petition or donate immediately after watching the video. Petitions can help NGOs to exert pressure on decision-makers to address the situation they highlight, and donations help them to continue their work in the longer run. As these videos likely generate strong negative emotional reactions, those who see them may use petitions and donations as a way of relieving their negative emotional state. As such, NGOs can use undercover videos to generate negative emotional reactions, and so push viewers towards actions immediately afterwards to alleviate these negative emotions.

We here empirically investigate three research questions regarding undercover videos. First, whether these videos successfully generate immediate engagement among viewers. Second, whether this increased immediate engagement reflects the viewers’ emotional reaction to the video: we focus here on the role of primary emotions (anger, fear, joy, surprise, disgust, and sadness), which are known to be instinctive, basic universal emotions that are experienced after a stimulus^6,7^. Third, whether activists anticipate this role of emotions in the immediate engagement that follows the viewing of the video.

The assessment of the role of emotions in reactions to undercover videos presents an empirical challenge. Observational data do not allow researchers to identify the mediated treatment effect of emotions (i.e. the greater engagement due to the change in emotions), as those who are exposed to the video are affected both by the video itself (the direct treatment effect) and the change in emotions resulting from the video (the mediated treatment effect). As such, observational data cannot predict how individuals would have reacted without having been emotionally affected. We here build an empirical model that draws from recent advances in the mediation-analysis literature^8–10^. We capture the mediated treatment effect by generating exogenous variations (experimental manipulation) on the mediator for participants exposed to the treatment. Our model estimates the mediated treatment effect by assessing to which extent the total treatment effect is affected by the exogenous changes in the mediator.

We design an experiment to elicit the role of primary emotions in the engagement generated by undercover videos in the context of animal advocacy. Animal-rights activists make intensive use of undercover videos to publicly criticize intensive farming and abbatoirs^11–13^. While public opinion largely supports the protection of animal welfare (e.g., over 80% of EU citizens say they care about farmed-animal welfare), individuals underestimate the prevalence of intensive farming and animal suffering^14^. Animal-advocacy NGOs see undercover videos as an effective way of providing information about what happens in intensive farms (that individuals cannot see) and countering the narratives put forward by the industry (e.g., via adverts). This strategy seems to have been effective, at least in the short term, as the petitions linked to these videos often attract hundreds of thousands of signatures.

In our experiment, participants are randomly exposed to an undercover video that was recorded on an intensive farm in France in 2021. In addition, subjects in the treated group are randomly assigned to a short or long version of the experiment. In the long-treatment arm, we introduce a 5-minute cooling-off period during which subjects answer a survey that is unrelated to animal welfare (covering travel preferences). Our pilot data showed that exposure to the video significantly affects individuals’ engagement in animal welfare, and generates strong negative emotions. The pilot data also showed that the cooling-off period significantly reduces the negative emotions arising from the video, which enables us to estimate the mediated treatment effect. Our main sample consists of N=3,310 participants. Ex-ante power analysis with the pilot data indicates that our experiment allows us to detect any mediated treatment effect of 3.4 percentage points or larger with a probability of at least 95%.

We analyze participants’ engagement through a charity-giving game in which individuals can give real money to four NGOs or three petitions in favor of animal welfare. Participants decide under a veil of ignorance (i.e. they ignore which NGOs will actually be selected in the charity-giving game) and report their donations for each contingency. We aggregate the giving decisions to construct a general score of pro-animal engagement (Cronbach’s alpha=0.87). Regarding emotions, we construct a negative-emotions score using self-reported information on six primary emotions: anger, disgust, fear, sadness, happiness, and surprise^15^. The general score of negative emotions is given by the sum of these emotions, with the happiness score being reverse-coded (Cronbach’s alpha=0.84). Based on the theoretical framework that we describe below, we hypothesized that the video would increase engagement and that emotions would play a significant role in this greater engagement. Accordingly, we first test whether the video successfully increases engagement, i.e. whether the total treatment effect is positive ($H_0^1: \Delta _{TT} \le 0$). Second, we test whether the emotional load generated by the video generates greater engagement, i.e. whether the mediated treatment effect of emotions is positive ($H_0^2: \Delta _M \le 0$).

Our registered analyses show a statistically significant positive effect of watching the video on donations (+3.81 percentage points in average donations, one-sided test: p$<0.001$). The two-sided 95% confidence interval indicates an average increase in donations comprised between +1.74pp and +5.93pp. However, we fail to reject the null hypothesis for the mediated treatment effect (one-sided test: p=0.175) and, therefore, cannot conclude that the negative emotional load plays a significant role in the video’s increase in donations. Nevertheless, on the other hand, our estimates cannot rule out the possibility that emotions play a role either. The mediated effect is estimated to be positive but of limited size (average estimate: +1.27pp), and the two-sided 95% confidence interval comprises values between −1.28pp and +3.96pp. While negative emotions sharply increase after watching the video, the failure to reject the null is driven by the fact that most of the increase in donations associated with the video survives the disappearance of the negative emotions when we introduce the cool-off. Our exploratory analyses show that the video mostly generates sadness, anger, and disgust, and decreases happiness among the viewers. They further suggest that more moderate actions benefit the most from the video (i.e., a ban on intensive farms, development of plant-based menus in university canteens, and support for farmers’ initiatives to improve animal welfare).

Last, we compare our results to the expectations of activists in the NGO that released the undercover video used in our experiment. Understanding activists’ expectations regarding the impact of their videos is important to decipher their strategy. Activists often claim that the emotional reaction only serves as a starting point and that they hope to stimulate a rational reaction with lasting effects. We analyze in a prediction study whether activists believe that the effect of the video on the general population will be substantially driven by emotions. We recruited 154 engaged activists via the NGO’s social-media groups for activists who were blind to the study’s hypothesis, and 50 completed the prediction study. We elicit their beliefs about the emotional impact of the video using a survey that was administrated during the data-collection period in the main experiment. We had no hypotheses regarding the views of activists before data collection, and we therefore carry out exploratory analyses only. The exploratory analyses suggest that activists tend to overestimate the altruistic behaviors of the general population. The median expectation among activists anticipates an increase in negative emotions and donations after watching the video but the emotional load is expected to last longer than what we observe.

The remainder of the paper is organized as follows. “Background” discusses the empirical and theoretical background, and “Methods” introduces the methods (ethics, design, and procedures). “Pilot data” presents the pilot data. “Analysis plan” then describes the analysis plan (empirical model, hypotheses), and “Sampling plan” the sampling plan. “Results” presents the results of the main experiment, and “Perception by activists” the prediction study with the activists. Last, “Discussion” discusses the results and concludes.

## Background

In recent years, activists across various causes have used undercover videos to raise awareness about social problems. Environmental NGOs regularly publish undercover videos exposing private companies that pollute the environment (e.g., Greenpeace on deep-sea mining, and Mother Nature on ecosystem pollution). Undercover videos are not restricted to environmental NGOs, and are also used by human-rights activists to denounce human-rights violations. For example, a number of NGOs have used undercover videos to reveal official corruption in their countries (e.g., the PCUD in Guinea) and torture in prisons (e.g., Public Verdict in Russia).

**Figure 1:**
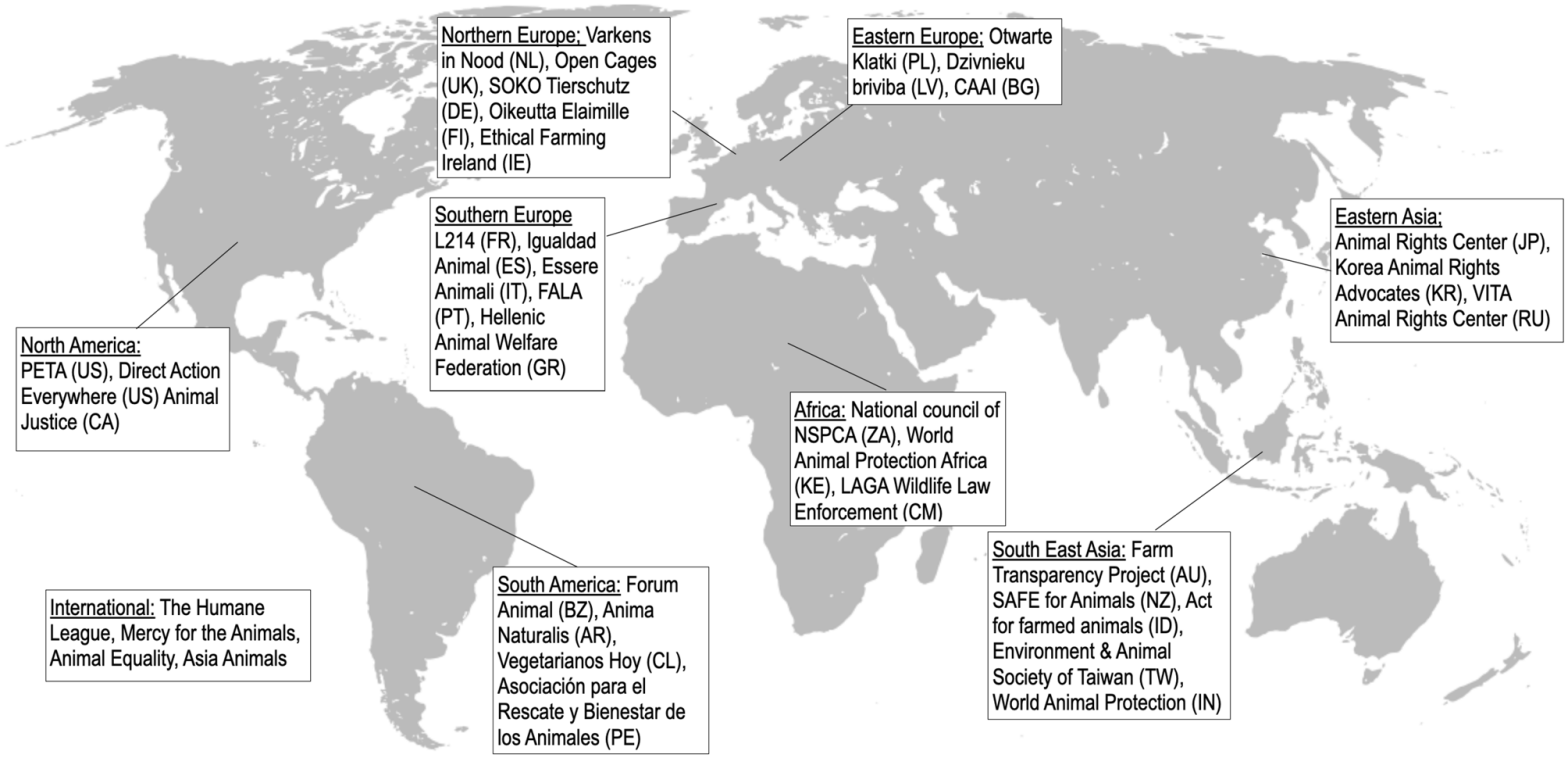
World map of animal-advocacy NGOs using undercover videos (non-exhaustive) Original map credits: San Jose. License: CC BY-SA 3.0 via Wikimedia Commons. Modified by the authors with Keynote (v 12.2.1). [https://commons.wikimedia.org/wiki/File:World_map_blank_gmt.svg?uselang=en].

Animal-advocacy groups have become one of the most-intensive users of undercover videos worldwide. Figure 1 below plots a non-exhaustive map of animal-advocacy NGOs that have published undercover videos (own data collection). NGOs using undercover videos are present in many countries (e.g., the Humane League and Peta in the United States, Animal Justice Project and Open Cages in the UK, Varkens in Nood in the Netherlands, Oikeutta Elaimille in Finland, and the Farm Transparency Project in Australia). Some NGOs are specialized in the publication of undercover videos. For instance, the US NGO “Mercy for Animals” has released 77 undercover videos in the past decade, and the US-based NGO “Animal Equality” reported 17 undercover investigations in 2021 only [See Note 1 in the Supplementary Materials]. Some NGOs like Sinergia Animal have become experts in publishing these videos, and operate worldwide.

The release of undercover videos showing animal abuse has also become very frequent in France, with L214 being one of the most-active NGOs worldwide. The French farming industry is one of the largest in the European Union, and most chickens (about 80%) and pigs (about 95%) are raised without outdoor access, i.e., in what activists consider as intensive farms. L214 has over the past decade become the largest NGO denouncing animal abuse on French farms. Since its creation in the early 2000s, L214 has released over 100 undercover videos. These videos denounce current farming conditions and regularly create significant citizen engagement. The petitions associated with these undercover videos often obtain over 100,000 signatures (e.g., a petition to close an abattoir—146,000; a petition for a Parliamentary investigative committee into abattoirs—145,000; petitions against intensive farming for chicken—167,000, and for pigs—148,000). [See Note 2 in the Supplementary Materials].

The rise of undercover videos among animal-advocacy groups likely reflects the substantial engagement that they create. However, the role of emotions in this engagement remains an open empirical question. In a recent interview (2022), the co-founder of the L214 NGO, Sébastien Arsac, declared that activists seek to stimulate engagement through two channels: “This emotion is a starting point. Any association that wants to alert and raise awareness of a serious subject will accompany the images with matching music. Then there is rationality: in our communication, we provide scientific analyses, comments from veterinarians, and we question the figures that are provided regarding the number of animals” [See Note 3 in the Supplementary Materials].

Assessing the role of emotions in the reaction to the videos is key to understanding the success of NGO campaigns. Evidence from Behavioral Sciences suggests that emotions are likely to be a major element of the greater engagement after watching the videos. A large body of research in Psychology indicates that emotions are powerful and pervasive drivers of beliefs and decisions^16^, with various applications to (for example) Marketing^17^ and Management^18^.

The negative emotional state generated by the videos is likely to be driven by negative primary emotions. The Psychological literature on emotions distinguishes primary emotions such as anger or sadness from secondary emotions such as guilt^19–21^. Showing animal mistreatment in intensive farms presents these animals as victims or “harmed patients”^22^, which probably increases the perception of their sentience and elicits negative moral emotions such as sadness towards the “patient” and anger towards the “agent”^23^. These videos likely generate strong primary negative emotions, as it has been shown that many individuals respond immediately after watching the video by signing petitions, for instance. These emotions have typically been shown to be short-lived. For example, facial expressions and physiological responses fade quickly over a short period of time^16,24^. Previous work has also shown that the emotional response following an anger-inducing trigger disappears after 10 min again suggesting a short primary-emotion lifespan^25^. One important question is then whether the immediate engagement generated by the videos is driven by these short-lived emotions.

Two main theories have tried to explain the effect of emotions on decision-making: the affect-as-information theory^26,27^ and the appraisal-tendency framework^28–30^. While both theories acknowledge the role of emotions in judgments and decision-making, they differ in their approach to how emotions are integrated. The affect-as-information theory posits that people usually rely on their general emotional state to make judgments and decisions. In other words, this theory suggests that people may make different decisions depending on their emotional state, as it influences their perception of a situation. For instance, when watching an undercover video of animal abuse, people may be more inclined to take action due to their heightened emotional state, but this effect may fade once the emotions generated by the video disappear.

On the other hand, the appraisal-tendency framework posits that different stimuli can trigger different appraisals, such as harm or unfairness, that can subsequently trigger different emotions, such as sadness or anger, and influence subsequent judgments differently. In other words, this theory suggests that emotions result from cognitive evaluations of situations. According to this framework, people may experience anger when perceiving an injustice - while they would consider the situation unjust because they experience anger according to the affect-as-information theory. In the case of undercover videos, the appraisal-tendency theory would predict that negative emotions arise from the perceived injustice of animal abuse. In that case, increased engagement in animal welfare may persist beyond the immediate emotional load induced by the video. Emotions and appraisals tend to have a reciprocal relationship, though, each reinforcing the other^31^, making it challenging to identify which one initiates the cycle.

While NGOs might attempt to provoke rational reactions to the videos, they may also have an interest in producing emotional reactions. Communication based on emotions is a key tool used by NGOs, via advertising campaigns and social-media channels^32,33^. A number of contributions have revealed the importance of emotions in various domains of political action. For example, emotions significantly affect the donations individuals make to NGOs^34–36^. It is therefore possible that activists predict a positive role for emotions in the increased support for their cause, and take advantage of this effect.

However, there are also opposing arguments suggesting a limited emotional impact of undercover videos. In the field of psychology, a substantial body of theories and research, spanning from psychoanalysis to social psychology, has proposed that individuals may engage in perceptual, cognitive, and behavioral distortions as a means of shielding themselves from the potentially distressing emotional responses that could arise in the absence of these defense mechanisms^37^.

In the domain of animal welfare and meat-eating, a large body of behavioral research has explored the complex psychology of consumers^38,39^. In particular, previous work has discussed the evidence for cognitive dissonance, also known as the “meat paradox”^40^. This paradox explains why omnivores and vegetarians have different beliefs about and attitudes towards animals^41,42^ and the willful ignorance about the treatment and the suffering of farmed animals^43–46^. Even when confronted with compelling evidence, people often demonstrate resistance to assimilating new information in this domain. For instance, it has been shown that people do not seem to process relevant information (e.g., animal intelligence) when it applies to an animal that they consume (e.g, pigs)^47^. Given these insights, it is plausible that viewers of undercover videos employ protective strategies (e.g., by distancing themselves from the animals, or engaging in biased information processing about their rearing conditions) to mitigate the emotional impact they experience.

## Methods

### Ethics information

This work received the approval of the ethics committee of the Institute for Advanced Studies in Toulouse in May 2022 (IRB approval number: 2022-04-001). It complies with all relevant ethical regulations for research with human participants. All of the participants were recruited through a representative sampling survey company called MSI and were compensated financially by the survey company for their participation in accordance with local standard rates. They received a fixed amount of money for their participation (a show-up fee) and a variable amount determined by their decisions (see the Design and procedure section). Participants were anonymous and were selected from the survey company’s database. They were randomly allocated to one of the four conditions described below. The sample is representative of the French population.

### Design and procedure

#### Design

The experiment is a between-subject randomized-control trial with equal randomization of participants across treatment branches. Figure SM1 in the Supplementary Materials provides a graphical representation of the course of the experiment.

We start by informing participants about the experiment, and tell them that we will ask a series of questions about their dietary habits, and their beliefs and attitudes towards public topics. Participants are also told that the survey will last about 10 min (20 min) if they are in the short condition (the long condition). Importantly, all participants are told that they may be exposed to explicit images during the study, and have the possibility of opting-out at this early stage of the experiment. For quota purposes, i.e. to have a balanced number of men and women, we ask participants their gender at this stage.

Participants next face a series of screens, the order of which is randomized with equal probability. On one screen, we ask participants the frequency with which they eat a series of food products (red meat, white meat, fish, eggs, dairy, vegetables, pulses, fruits, and starchy products). We further ask them whether they identify as carnist, omnivorous, flexitarian, vegetarian, or vegan. Participants are then asked to test the sound of their computer by listening to a 4-s sound of a grasshopper and say what type of sound they have just hear, from a list of five (a grasshopper, a car alarm, a meowing cat, a whistle, and a barking dog). The study ends here for participants who provide incorrect answers.

There are variations in treatment in the following screens (see Table 1 below). Half of the participants are randomly exposed to a video, and the other half not. The video is a one-minute and twenty-eight-second extract from a video published online by a French animal welfare NGO (L214), and shows footage from a French pig farm in 2021. The name of the NGO is not displayed in the extract. The video contains some written comments in French. The video shows pigs’ living conditions in a French intensive farm (e.g., no outdoor access, high density, stabling, sow farrowing crates, killing piglets by hitting them against a hard surface). For participants in the treated group, we record the time spent watching the video, and the number of clicks.

Second, participants are also randomized between two other treatment arms. Half are randomly assigned a short version of the survey, and now proceed to the next series of questions below. The other half are assigned to a long version of the survey, where they answer a series of questions about their travel preferences (train vs. plane). The objective of the long survey is to produce a cooling-off period to mitigate the emotional reaction generated by the video. The questionnaire about travel preferences appears in the Supplementary Materials. Overall, participants in the experiment are allocated with equal probability to one of the four conditions: no video and short survey (Novid-Short), no video and long survey (Novid-Long), video and short survey (Vid-Short), and video and long survey (Vid-Long).

**Table 1:** Treatment-assignment probabilities.

	Without video	With video
Short survey	Novid-Short	Vid-Short
	25% of the sample	25% of the sample
Long survey	Novid-Long	Vid-Long
	25% of the sample	25% of the sample

After the treatment, we ask respondents to report their emotions. They first indicate the extent to which facial expressions of six primary basic emotions (anger, disgust, fear, sadness, happiness, and surprise) best reflect their emotional state. This face-emotion scale consists of three male and three female faces from the well-validated Radboud Faces Database for each emotion^15^, which has been widely used as an alternative to verbal self-reports^19,20^. Participants first click on the set of faces that best match how they felt while watching the video. They then rate how well each set of faces matches their current feelings on a scale from 1 (strongly disagree) to 7 (strongly agree). The order of these sets of faces is randomized at the individual level with equal probability.

Next, all participants can donate some money to improve animal welfare. They are told that they will receive an additional amount of 1 Euro for their survey participation that they can either keep or donate (fully or partially). We introduce a list of four French NGOs and three petitions related to animal welfare to which the participants can donate (for the latter, they donate to increase the petition’s visibility). They decide under a veil of ignorance and indicate how much they would like to give for each donation option knowing that only one of their seven donation decisions will be implemented and that the effective recipient of the donations was selected before data collection. The NGO list includes NGOs working for the development of plant-based menus in university canteens (Assiettes Végétales), fighting against intensive farming and the consumption of animal products (L214), fighting against the abandonment of pets and facilitating adoption (SPA), and supporting farmers’ initiatives aimed at improving farm-animal welfare (Welfarm). The three petitions are, respectively, against intensive farming, supporting plant-based meals in public canteens, and protecting animal-welfare activists who record videos in intensive farms.

Participants then reply to a series of questions on the next screens that assess their moral concerns for animals, using the questions developed in previous work^48^. We further elicit participants’ pro-meat justifications via a list of statements used in previous work^49^, and their motivation to eat meat right now to assess any potential reactance. We next ask participants who were exposed to the video whether they consider the farming practices shown in the video to be morally wrong and whether they would share the video on Twitter or Facebook if they had seen it on these social platforms. We further ask them a series of questions about their perception of the truthfulness and credibility of the video: whether they think that (i) the video is representative of animal farming in France, (ii) it was not recorded in France, (iii) it is a fake video made up by activists, (iv) the information provided in the video is not exact, (v) some pieces of information are distorted or biased, (vi) the video is not credible, and (vii) the video is not realistic.

The last part of the questionnaire includes control checks and demographic questions. We ask participants to indicate the topic of the study (i.e., diets), which we explicitly mentioned at the beginning of the survey. Second, participants in the video conditions were asked about the contents of the video (via a multiple-choice question), and whether they had seen this video before, or a similar video before. Third, all participants underwent an attention check (a simple calculation exercise). Last, we asked demographic questions (age, marital status, parenthood, political orientation, and religion).

#### Outcome variables

We consider two outcome variables to measure the effect of the different treatments. We first create a score of the propensity to engage in animal welfare. This pro-animal score is the average of the donations in cents that are made to the NGOs and petitions divided by 100 and so takes on values between 0 and 1 with higher values reflecting a larger endowment share donated to animal welfare. Second, we investigate the role of emotions by generating an aggregate negative-emotion score. We take the sum of all emotions (with happiness reverse-coded). We standardize this average figure by adding two and dividing by 36, yielding a negative-emotion score varying between 0 and 1, with higher scores reflecting more negative emotions [See Note 4 in the Supplementary Materials]. This negative-emotion score aims to assess the negative emotional state of participants by considering the intensity with which they experience different primary emotions that have different valences (happiness has a positive valence and the remaining emotions have a negative valence).

#### Outcome-neutral tests

We committed to assessing the validity of our analysis by running the following outcome-neutral tests. We first committed to an estimation of the Cronbach’s alpha figures for both outcome variables. If these are above 0.6, we committed to saying that our outcome variables have sufficient internal validity. Second, we committed to reporting whether the cooling-off period reduces the negative-emotion score by at least 20 percentage points (Vid-Short vs. Vid-Long). If not, we we committed to considering that the protocol does not generate sufficient variation in emotional states to estimate the mediated treatment effect. Third, we set up an outcome-neutral test with respect to attrition between treatments. We committed to comparing the distributions of participants at the beginning of the survey (after gender quota, consent validation, and sound test) and at the end of the survey (complete answers, validation of the attention checks). We committed to considering attrition to be a concern for the validity of the experiment if the share of participants changes by more than 10 percentage points in at least one treatment arm. If one of these three outcome-neutral tests fails, we committed to mentionning that our estimates carry little weight and should be interpreted with caution.

Last, we also committed to testing whether the Long questionnaire generates *per se* changes in donations. We committed to running a wilcoxon rank-sum test on the aggregate donation score testing equality between Novid-Short and Novid-Long. If the test rejects the null hypothesis (i.e., equality) at $\alpha =5\%$ (i.e., the difference is statistically significant) and if the absolute difference exceeds 2 percentage points (i.e., the difference is economically significant), we committed to correcting our estimates using the estimator described in the Supplementary Materials.

#### Exclusion rules

We drop incomplete observations, including participants who did not give their consent and who did not complete the experiment. Second, we drop participants who failed the sound test (to ensure that respondents can hear the sound of the video). Third, we drop participants who failed the attention checks (they have to say that the study is about diet, answer correctly regarding the content of the video, and complete the simple calculation task). Last, we also drop participants who spent less than 90 seconds on the survey (excluding the time spent watching the videos for the individuals in the video-treatment groups).

## Pilot data

We carried out an online pilot experiment on 568 French participants; this was conducted online by the Dynata research panel company. Data collection took place from June 21st 2022 to June 24th 2022. A total of 905 participants were recruited: 442 were randomly allocated to the Short questionnaire and 463 to the Long questionnaire. Among these respondents, 9 did not give their consent and were excluded at the very beginning of the questionnaire, and 135 were not invited to continue with the questionnaire in order to produce a gender-balanced sample. We dropped participants who reported the incorrect sound, who did not finish the questionnaire, and who failed the attention checks [See Note 5 in the Supplementary Materials]. The final sample consisted of 568 respondents (220 women; average age $=$ 45.3, SD $=$ 13). Of these, 279 completed the Short questionnaire, with 133 exposed to the video (Vid-Short) and 146 to the control condition with no video (Novid-Short), and 289 completed the Long questionnaire with 136 exposed to the video (Vid-Long) and 153 to the control condition with no video (Novid-Long).

**Figure 2:**
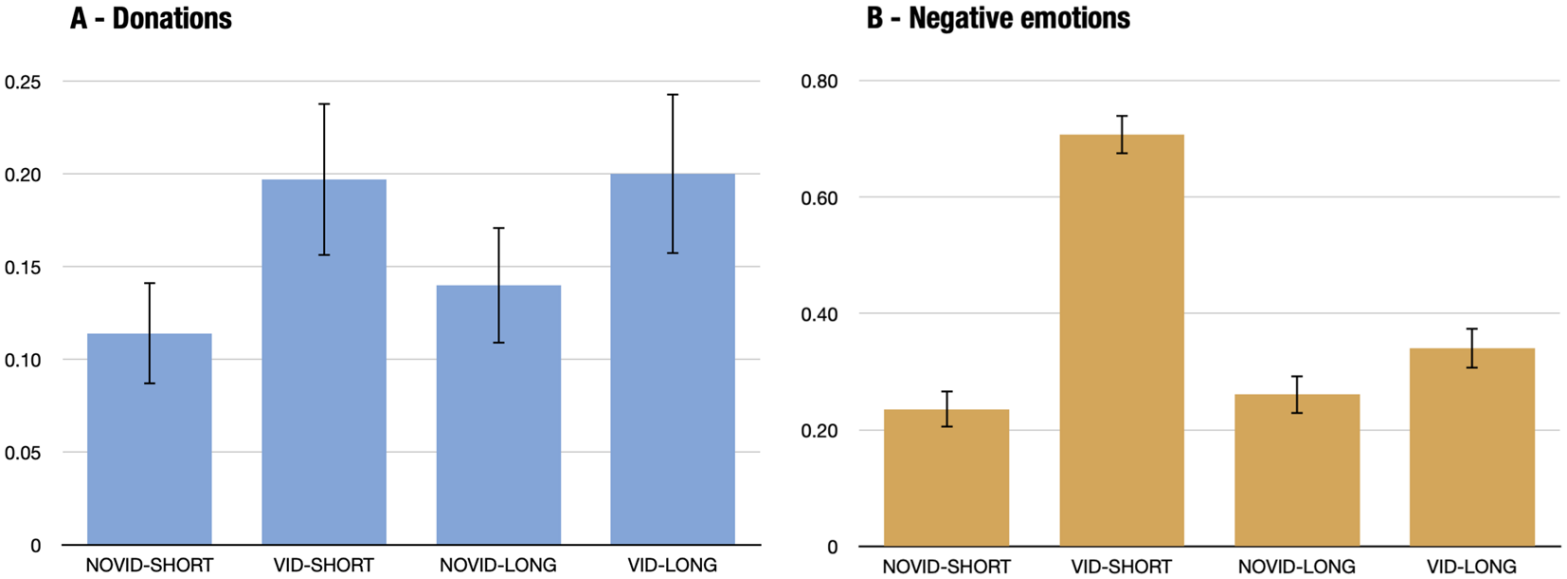
The differences in donations and negative emotions in the pilot study by treatment condition The error bars show the 95% confidence intervals. Overall sample size $N=568$. (**A**) The donation score is the normalized average of donations to all NGOs and petitions. (**B**) The negative-emotion score is the normalized average of all emotions (with happiness reverse-coded).

The pilot-study results indicate that our indicators perform well. The Cronbach’s alpha’s for the two outcome variables are 0.90 (donation score) and 0.89 (negative-emotion score). Figure 2 also shows that the video has a strong impact on donations and emotions. We further observe that the Long treatment variation significantly mitigates the negative emotions that arose from watching the video, but does not affect average donations. There is a large share of zero donations: in the Short questionnaire: 38% of participants do not give anything when they do not see the video, and 26% after watching the video. This suggests that the treatment can have a significant impact on the extensive margin (i.e. whether to donate or not). Last, we can note that cooling-off does not affect donations in the absence of a video (Novid-Short vs. Novid-Long: $p>0.477$ for all of the single-donation decisions).

## Analysis plan

### Empirical model

Our objective is to estimate the overall effect of undercover videos on individuals’ decisions to engage (the total effect), and more specifically the role of immediate emotions in this effect (the mediated effect). The total effect can easily be estimated by comparing engagement across treatments (with vs. without exposure to the video). Assessing the role of emotions is more complex. In what follows, we estimate the mediated (or indirect) treatment effect by building an empirical model that relies on recent advances in the field^8–10^. Observational or experimental data with a single treatment variation (one control and one treated group) typically fail to correctly estimate the mediated treatment effect, as the mediator and the outcome variables are both affected by the treatment. As such, observational data cannot distinguish the changes in the outcome variable for the treated that are due to the treatment status (the unmediated treatment effect) from those that are due to a change in the mediator (the mediated treatment effect). The introduction of a cooling-off period in our experiment allows us to disentangle these two effects.

We denote by $Y_i$ the amount of money individual *i* gives to the animal-welfare charities, and $M_i$ her emotional state. We define four potential outcomes $Y_i^{d,m}$ that depend on whether the individual saw the video ($d=1$) or not ($d=0$), and on her emotional state which can be high ($m=1$) or low ($m=0$). We further define two potential values for the mediator $M_i^d$, depending on whether the individual was exposed to the video ($d \in \{0,1\}$). Last, we note $D_i$ the dummy variable equal to 1 if the individual is in the treated condition (with video), and 0 otherwise (no video).

Following the classic typology in causal analysis^50^, we consider four types of participants depending on their emotional reaction to exposure to the video^10^. First, always takers are individuals who are always in a high emotional state ($M_i^1=M_i^0=1$): we denote them by $T_i=a$. Second, never takers ($T_i=n$) are, on the contrary, always in a low emotional state ($M_i^1=M_i^0=0$). Third, compliers ($T_i=c$) are assumed to have a low emotional state in the absence of the video ($M_i^0=0$) but a high emotional state after they watch it ($M_i^1=1$). Last, the canonical framework also allows for defiers ($T_i=f$): individuals who have opposite reactions to those of the compliers ($M_i^0=1, M_i^1=0$). Here defiers would be individuals who enter the experiment with a high emotional state but become more relaxed after watching the video of animal abuse. We consider it very unlikely that any participants (or only a very few of them) would feel relieved by watching a video showing animal abuse in intensive farms. We thus assume $T_i \in \{a,n,c\}$. We denote by $p_a$, $p_n$, and $p_c$ the respective shares of always takers, never takers, and compliers in the population.

The total effect of the treatment on the treated is given by:

1 $$\begin{aligned} \Delta _{TT}={\mathbb {E}}[Y_i^{1,M_i^1}-Y_i^{0,M_i^0}|D_i=1] \end{aligned}$$

As we have random assignment of the video, ${\mathbb {E}}[Y_i^{0,M_i^0}|D_i=1]={\mathbb {E}}[Y_i^{0,M_i^0}|D_i=0]$. We can then rewrite the previous equation as:

2 $$\begin{aligned} \Delta _{TT}={\mathbb {E}}[Y_i^{1,M_i^1}|D_i=1]-{\mathbb {E}}[Y_i^{0,M_i^0}|D_i=0] \end{aligned}$$

The total effect of the treatment on the treated can thus be estimated as the difference in average donations between the Vid-Short-Short and Novid treatments.

We define the mediated treatment effect of the video ($\Delta _M$) as the change in donations associated with the change in the emotional state conditional on seeing the video. This is:

3 $$\begin{aligned} \Delta _M={\mathbb {E}}[Y_i^{1,M_i^1} - Y_i^{1,M_i^0} |D_i=1] \end{aligned}$$

We can decompose this by types of individuals, which yields:

4 $$\begin{aligned} \Delta _M&=p_a \times {\mathbb {E}}[Y_i^{1,1} - Y_i^{1,1} |D_i=1, T_i=a] \nonumber \\&+p_n \times {\mathbb {E}}[Y_i^{1,0} - Y_i^{1,0} |D_i=1, T_i=n] \nonumber \\&+p_c \times {\mathbb {E}}[Y_i^{1,1} - Y_i^{1,0} |D_i=1, T_i=c] \nonumber \\ \Delta _M&=p_c \times {\mathbb {E}}[Y_i^{1,1} - Y_i^{1,0} |D_i=1, T_i=c] \end{aligned}$$

The cooling-off period aims to manipulate the mediator among the compliers who see the video. We denote participants exposed to the cooling-off period by $C_i=1$ and those who are not by $C_i=0$. The pilot data suggest that this cooling-off period significantly reduces participants’ emotional stress, although this does remain slightly higher than that in the absence of the video. We thus consider that the cooling-off period successfully manipulates the mediator for a share *q* of the compliers who saw the video but does not do so for a share of $1-q$. We assume that the treatment effect is the same for both types of compliers.

The expected difference in mean donations between participants in the Vid-Short and Vid-Long treatments is denoted by $\delta$ and given by:

5 $$\begin{aligned} \delta = {\mathbb {E}}[Y_i |&D_i=1, C_i=0] -{\mathbb {E}}[Y_i | D_i=1, C_i=1]= \end{aligned}$$

6 $$\begin{aligned}&p_a \times ({\mathbb {E}}[Y_i^{1,1}|D_i=1, C_i=0, T_i=a]-{\mathbb {E}}[Y_i^{1,1}|D_i=1, C_i=1, T_i=a]) \nonumber \\&+p_n \times ({\mathbb {E}}[Y_i^{1,0}|D_i=1, C_i=0, T_i=n]-{\mathbb {E}}[Y_i^{1,0}|D_i=1, C_i=1, T_i=n])\nonumber \\&+p_c \times \{ {\mathbb {E}}[Y_i^{1,1}|D_i=1, C_i=0, T_i=c]\nonumber \\&-(1-q){\mathbb {E}}[Y_i^{1,1}|D_i=1, C_i=1, T_i=c]-q{\mathbb {E}}[Y_i^{1,0}|D_i=1, C_i=1, T_i=c] \} \end{aligned}$$

We then assume that the cooling-off variation does not affect donations directly, but rather only through the mitigation of emotions. We found empirical support for this assumption in the pilot data, where adding a cooling-off period without the video (i.e. comparing Novid-Short to Novid-Short) did not affect donations or emotions. The expression above simplifies to:

7 $$\begin{aligned} \delta =p_c \times q \times {\mathbb {E}}[Y_i^{1,1} - Y_i^{1,0}|D_i=1, T_i=c] \end{aligned}$$

The mediated treatment effect is then given by:

8 $$\begin{aligned} \Delta _M=\frac{\delta }{q} \end{aligned}$$

Note that we provide an alternative estimator of the mediated treatment effect in the Supplementary Materials in case our experimental data show a significant change in donations between Novid-Short and Novid-Long (see outcome neutral tests).

The parameter $\delta$ can be estimated from the data by comparing average donations in Vid-Short and Vid-Long treatments. The parameter *q* corresponds to the share of compliers who return to $M=0$ after the cooling-off period. We denote $M_k^{d,c}={\mathbb {E}}[M_i|D_i=d,C_i=c,T_i=k]$. The parameter *q* can be estimated from the following equation:

9 $$\begin{aligned}&\frac{{\mathbb {E}}[M_i|D_i=1,C_i=0]-{\mathbb {E}}[M_i|D_i=1,C_i=1]}{{\mathbb {E}}[M_i|D_i=1,C_i=0]- {\mathbb {E}}[M_i|D_i=0]}\nonumber \\&\quad = \frac{p_a (M_a^{1,0}-M_a^{1,1})+p_n (M_n^{1,0}-M_n^{1,1}) +p_c (M_c^{1,0}-M_c^{1,1})}{p_a (M_a^0-M_a^{1,0})+p_n (M_n^0-M_n^{1,0}) + p_c (M_c^0-M_c^{1,0})} = q \end{aligned}$$

as $$M_a^{1,0}=M_a^{1,1}=M_a^{0}=1$$, $$M_n^{1,0}=M_n^{1,1}=M_n^{0}=0$$, $$M_c^0=0$$, $$M_c^{1,0}=1$$ and $$M_c^{1,1}=(1-q)$$.

#### Estimation procedure

In the final sample, we estimate $\delta$ by calculating the difference in the average donation scores between participants in the Vid-Short and Vid-Long treatments (i.e., $\hat{\delta }={\overline{Y}}_{\text {VS}}-{\overline{Y}}_{\text {VL}}$) [See Note 6 in the Supplementary Materials], and *q* as the difference in the average negative-emotion score between participants in the Vid-Short and Vid-Long treatments divided by the difference in the average negative-emotion score of participants in the Vid-Short and Novid-Short treatments (i.e., ${\hat{q}}=({\overline{M}}_\text {VS}-{\overline{M}}_\text {VL})/({\overline{M}}_\text {VS}-{\overline{M}}_\text {NS})$). The mediated treatment effect is estimated as the ratio of these two estimates.

### Hypotheses

We have two objectives. The first is to determine whether the undercover video successfully generates immediate engagement for the NGO’s cause among viewers. In other words, we test $H^1_0: \Delta _{TT} \le 0$. If we reject $H^1_0$, we will conclude that the undercover video successfully creates engagement. The second objective is to determine the role of emotions in the changes in engagement in favor of the NGO’s cause. Following the above procedure, we estimate the mediated treatment effect $\Delta _M$. The theoretical section suggests that emotions play a causal mediation effect in changing individual behaviors. We test this hypothesis via $H^2_0: \Delta _M \le 0$. If we reject $H^2_0$, we will conclude that emotions play a mediating role in the changes in pro-animal behaviors after seeing the NGO’s video. If we fail to reject $H^2_0$, we will calculate the one-sided 95% confidence interval to estimate the upper bound for the value of $\Delta _M$.

We committed to testing the two hypotheses by looking at the lowest bounds of the one-sided 95% confidence interval of $\Delta _{TT}$ and $\Delta _M$ via bootstrap estimation ($B=1,000$). The lower bound will be set at the 5th percentile of the bootstrap distribution. If the 5th percentile is strictly larger than 0, we will reject the associated null hypothesis. If $H^1_0$ is rejected, but not $H^2_0$, we will calculate the 95th percentile of the bootstrap distribution, which will correspond to the upper bound of the estimated $\Delta _M$. If the upper bound of $\Delta _M$ is estimated to be strictly lower than 3 percentage points, we will consider that emotions play an economically insignificant role in mediating the effect of the videos. Otherwise, we will conclude that the study is inconclusive [See Note 7 in the Supplementary Materials].

## Sampling plan

**Figure 3:**
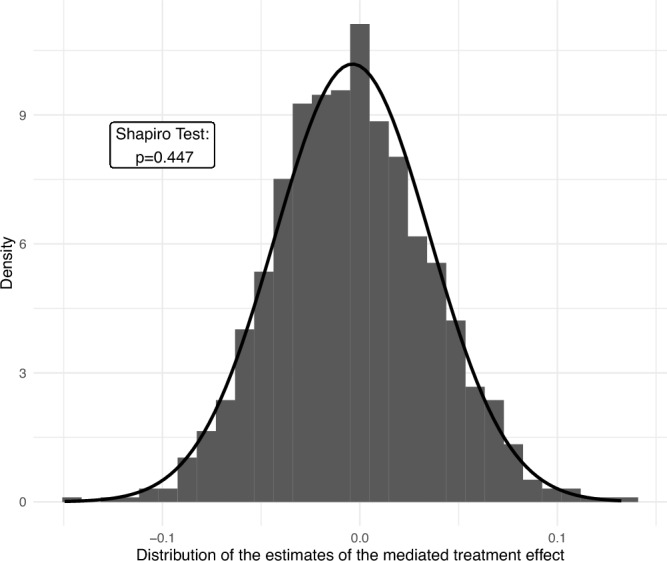
Bootstrapped distribution of the mediated treatment effect in the pilot data (B = 1000 draws).

We determined our sample size by assessing the precision of our estimates. We focused here on statistical power for the mediated treatment effect, as the mediation analysis requires more observations than does the estimation of the overall treatment effect. We considered that our analysis should be able to detect a mediated treatment effect of 5 percentage points or more with a probability of at least 95%, with a probability of Type-1 error equal to $\alpha =0.05$. Figure 3 shows that the bootstrapped distribution of the estimated $\Delta _M$ using our pilot data (with $B=1000$ draws) is approximately normal (Shapiro p-value = 0.447). Assuming that our estimate of $\Delta _M$ is normally distributed, the above decision rule for $H^2_0$ should be able to reject $H^2_0$ if the effect size is larger than 1.645 standard errors (i.e. the 5th percentile of a normal distribution). In this case, an estimated effect size of 0.05 (i.e. 5 percentage points) would lead to the rejection of $H^2_0$ if the standard error is smaller than 0.030.

Based on the pilot data, we simulated samples of different sizes using bootstrapping with replacement (assuming that the sample data are representative of the population data), and reported the precision of our estimate of the mediated treatment effect for each sample size. We simulated $S=1000$ samples for each sample size under consideration (with $B=1000$). The R code is provided in the Supplementary Materials. Note that, in this procedure, we did not use the effect size of the pilot data to compute statistical power as it would lead to a follow-up bias in study selection^52,53^. We relied here on the estimates of the standard errors and compute statistical power for an assumed effect size of 5 percentage points.

These simulations indicated that 800 observations per treatment would yield a 100% probability of rejecting $H_0$ if the true effect size is equal to 5 percentage points. We further estimated that the smallest effect that we can detect with a 95% probability with N = 3200 observations is 3.4 percentage points.

## Results

The experiment took place in December 2023 in accordance with GDPR regulation. Informed consent was obtained from all the study participants. In total, 3310 participants completed the survey, and passed the attention checks (women: 52%, average age = 47.8, SD=15.0) [See Note 8 in the Supplementary Materials]. Participants were randomly assigned to each of the four conditions with equal probability, and the final distribution of participants is displayed in Table 2. The experiment passed all the outcome-neutral tests. First, the Cronbach alphas are above the pre-specified 0.6 threshold for donations (0.87) and emotions (0.84). Second, the cool-off period reduced the emotional score by more than 20 percentage points. Third, the differences in attrition rates do not exceed the pre-specified 10-pp window. Fourth, we do not find any statistical effect of the cool-off per se when we compare donations in Novid-Short and Novid-Long (Wilcoxon test: p = 0.141, difference in averages: 1.15pp). We thus proceed to the confirmatory analysis with the main estimator presented above.

**Table 2:** Distribution of participants with complete answers who passed the attention checks.

	Without video	With video
Short survey	Novid-Short	Vid-Short
	N = 858	N = 794
	25.9% of the sample	23.9% of the sample
Long survey	Novid-Long	Vid-Long
	N = 850	N = 808
	25.7% of the sample	24.4% of the sample

### Confirmatory analyses

**Figure 4:**
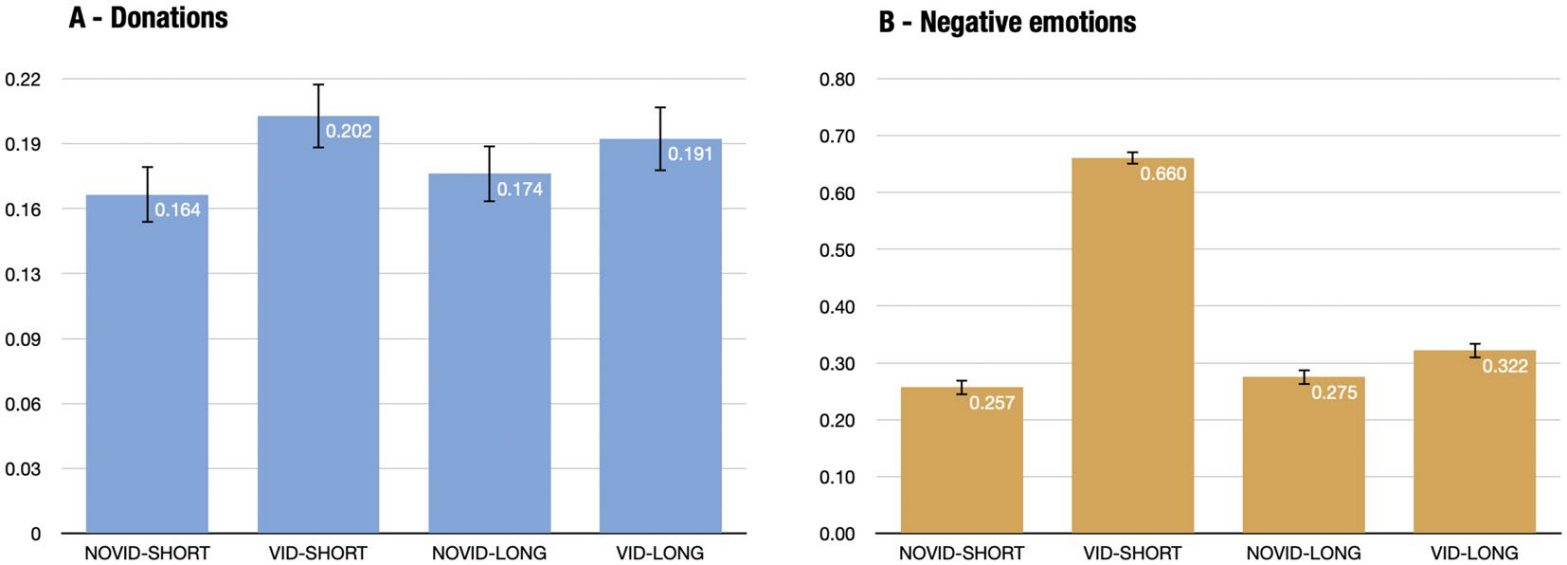
Average donations and emotions across treatments. Bars display average scores per treatment. Spikes represent 95% confidence intervals.

The main results of the experiment are displayed in Fig. 4. At first sight, we observe that the video increases average donations in the short questionnaire (from 16.4 to 20.2%) together with the average negative emotions (from 25.7 to 66.0%). The cool-off device is associated with a slight decrease in average donations (from 20.2 to 19.1%) and a large decrease in average negative emotions (from 66.0 to 32.2%).

**Figure 5:**
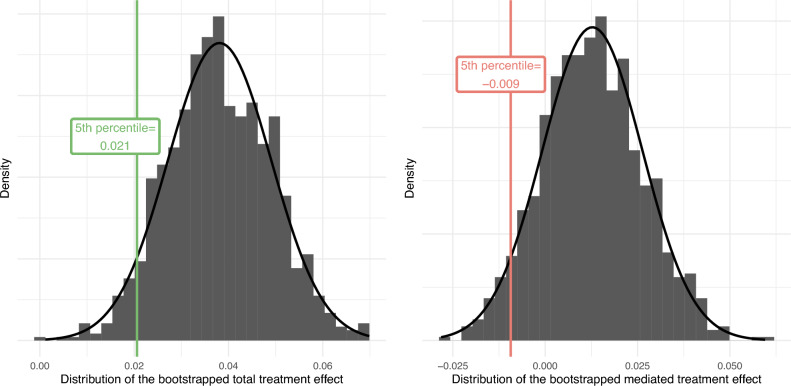
Bootstrap distributions of the total and mediated treatment effects with 1000 draws.

The bootstrap distributions of the estimates for the total and mediated treatment effects are displayed in Fig. 5. We rely here on our pre-registered decision rule to reject the null hypothesis, i.e., we reject the null if the 5th percentile of the bootstrap distribution of the estimator is strictly above 0. First, we can reject the null hypothesis for the total treatment effect (the 5th percentile is equal to +2.1pp), indicating that the video significantly increases donations to pro-animal actions (NGOs, petitions). The average effect (i.e., the mean of the bootstrap distribution) indicates an increase of 3.81pp. The p-value can be defined as the share of the distribution that is below 0, i.e., p$<0.001$. Given that the average donation score is equal to 16.4% in the control group (Novid-Short), this corresponds to an increase of 23.4% in donations. The lower and upper bounds of the two-sided 95% confidence interval are given by the 2.5th and 97.5th percentiles of the bootstrap distribution respectively, i.e., +1.74pp and +5.93pp.

Second, we find inconclusive evidence for the mediated treatment effect. On the one hand, we do not reject the null hypothesis of null or negative mediated treatment effect ($H_{0}^2: \Delta _M \le 0$, p = 0.175). The fifth percentile of the bootstrap distribution is equal to $-0.009$, falling below zero, and 17.5% of the distribution takes null or negative values. This provides evidence in favor of the null hypothesis (i.e., null or negative mediated treatment effect) and contradictory to the alternative hypothesis (i.e., strictly positive mediated treatment effect). On the other hand, we also fail to reject an economically significant effect of emotions (p = 0.108). Following our registered data analysis protocol, we investigate whether we can affirm that the mediated treatment effect is economically insignificant. To do so, we scrutinize evidence suggesting that the mediated treatment effect would be larger than +3pp, i.e. the registered threshold below which we consider the mediated treatment effect to play an economically insignificant role. We find that the 95th percentile is equal to 0.035, and about 10.8% of the bootstrap distribution is above the 3pp threshold. We are thus not able to reject the hypothesis of an economically significant role of emotions either. Altogether, we find an average small positive mediated treatment effect (+1.27pp increase in donations) but we do not have statistical support to rule out a null or negative mediated treatment effect ($\Delta _M \le 0$) of emotions nor to rule out an economically-significant effect ($\Delta _M \ge 0.03$). The two-sided 95% confidence interval is equal to $[-0.0128,0.0396]$.

### Exploratory analyses

**Figure 6:**
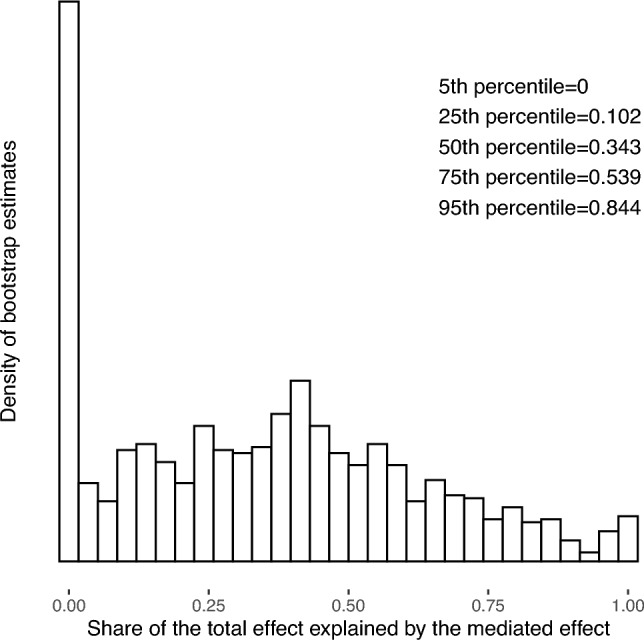
Bootstrap distributions of the share of the estimated total treatment effect explained by the estimated mediated treatment effect (1000 draws).

We now turn to some exploratory analyses that were not pre-registered. First, we explore further the total and mediated treatment effects. With the bootstrap draws, we can compute the share of the total treatment effect that could be explained by the mediated treatment effect. For each draw, we compute the ratio of the estimated mediated treatment effect over the estimated total treatment effect. When the mediated treatment effect is negative, we consider that it explains 0% of the total treatment effect. We winsorize this ratio to get maximal values at 1, which concerns about 1.4% of the estimates. Results are displayed in Fig. 6. Logically, 17.5% of the distribution is at 0 because 17.5% of the bootstrap distribution for the mediated treatment effect is lower or equal to 0 (see above). About half of our bootstrap estimates report that the mediated treatment effect explains one-third of the total treatment effect or less (i.e., median estimate equal to 34.3%). At the 95th percentile, the mediated effect accounts for 84.4% of the total treatment effect. The two-sided 95% confidence interval is equal to [0.00, 0.94], suggesting a wide range of values for the potential role of emotions in the increase in donations. Note that the particularly large confidence interval is due to the fact that taking the ratio of two estimates adds another layer of uncertainty, and our study was not ex-ante-powered to address this question. Overall, we find inconclusive evidence about the mediating role of primary emotions in the video’s impact on donations.

**Figure 7:**
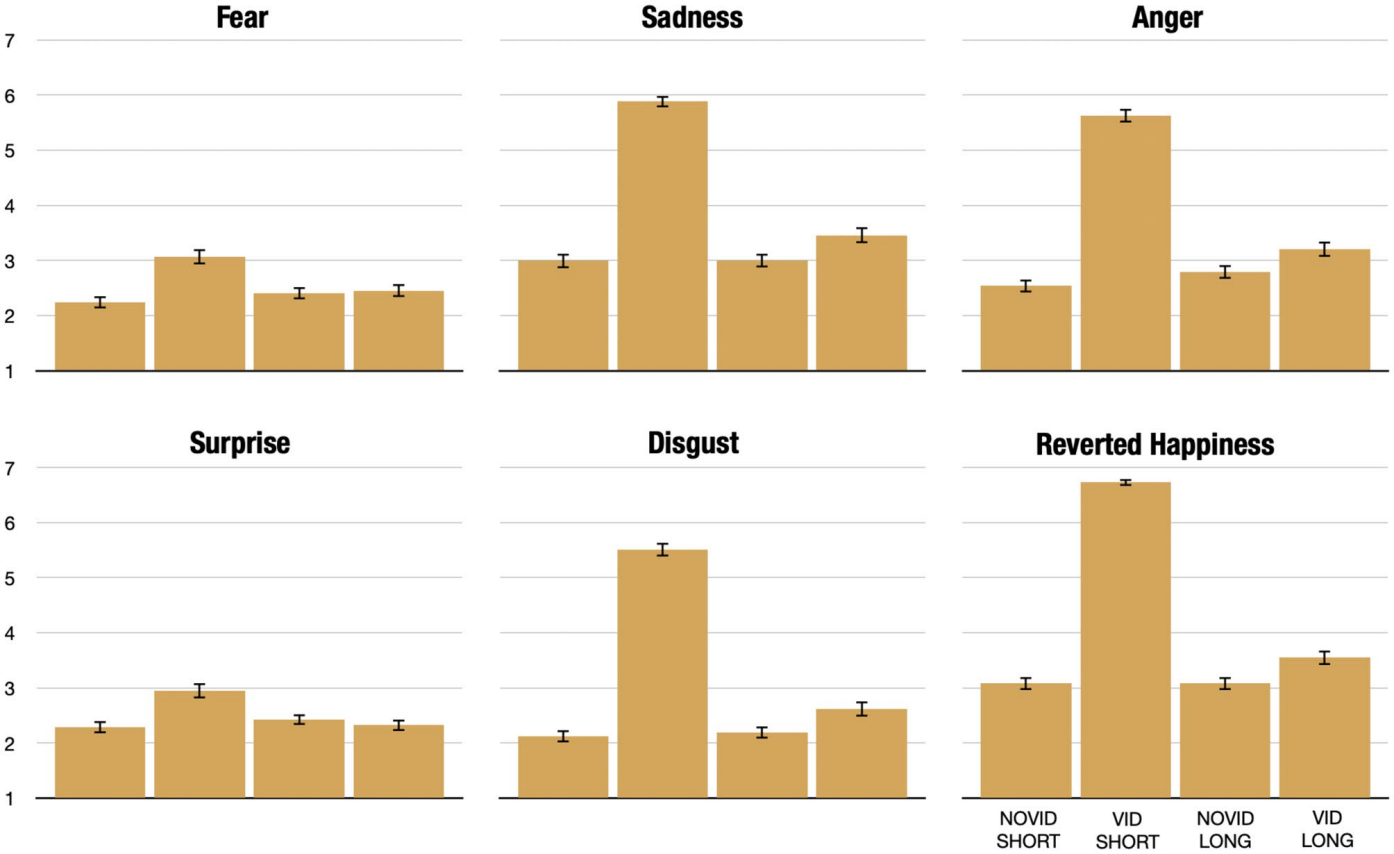
Average emotions per treatment. Bars display average scores per treatment. Spikes represent 95% confidence intervals.

Second, we look at how the video impacted single emotions (1-to-7 Likert scales). Figure 7 shows that the video mostly increased sadness (+2.89 between Novid-Short and Vid-Short, Wilcoxon p-value$<0.001$), anger (+3.09, p$<0.001$), and disgust (+3.91, p$<0.001$) scores and decreased happiness ($-3.65$, p$<0.001$). To a smaller extent, the video increased fear (+0.83, p$<0.001$) and surprise (+0.66, p$<0.001$) scores. The cool-off device appears to have mitigated this change in negative emotions for all emotions under consideration (for all emotions between Vid-Short and Vid-Long: p$<0.001$).

**Figure 8:**
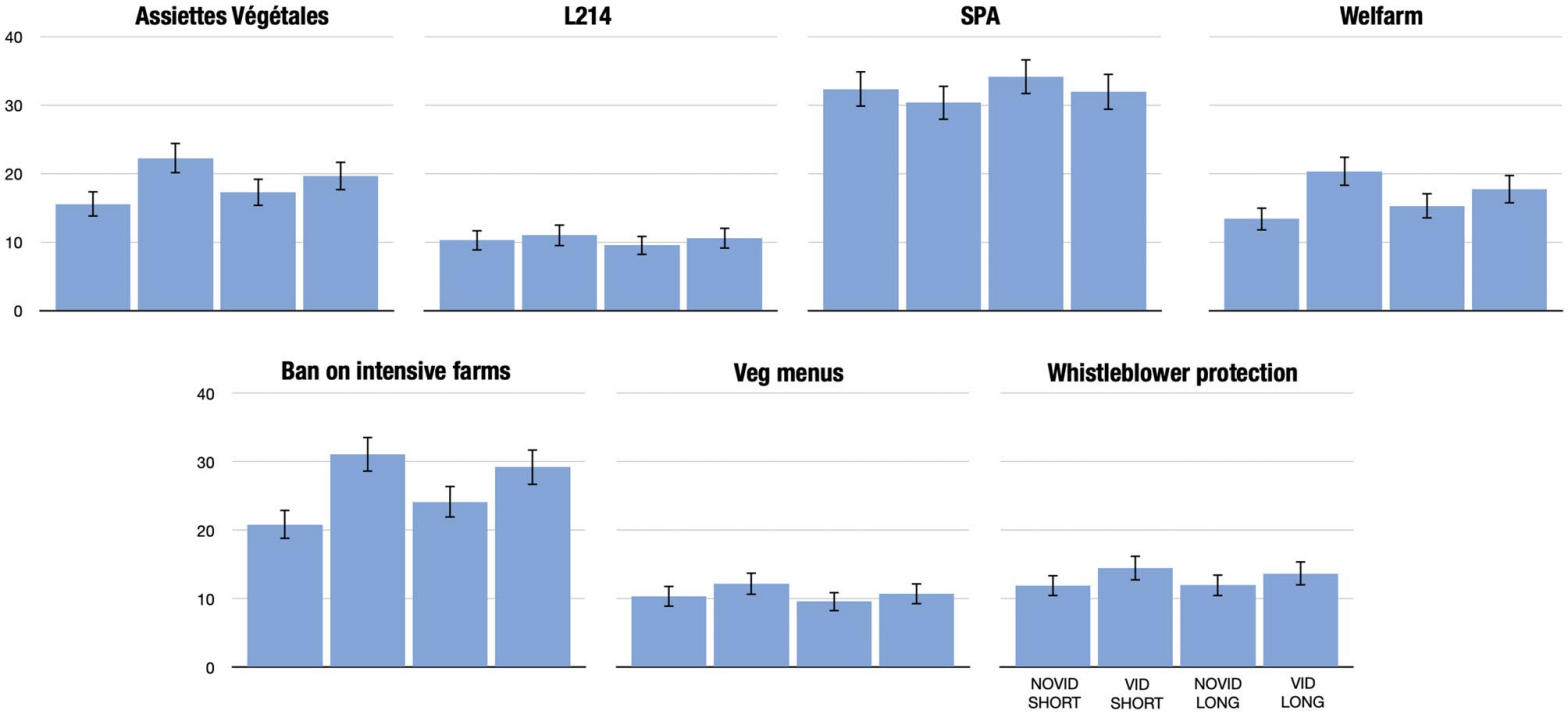
Average donations per treatment. Bars display average scores per treatment. Spikes represent 95% confidence intervals.

Third, we analyze the change in donations for each NGO and petition separately (Novid-Short vs Vid-Short). Average donations per treatment are displayed in Fig. 8. Exploratory non-parametric tests (Wilcoxon) suggest that the video had a statistically significant impact (p $<0.05$) on all donations but for two NGOs (L214, p = 0.052; SPA, p = 0.728). The largest increases in average donations were for two NGOs, i.e., *Assiettes Végétales* (+6.67pp, p$<0.001$) and *Welfarm* (+6.96pp, p $<0.001$), and for the petition demanding a ban on intensive farms (+10.22pp, p $<0.001$). These exploratory results suggest that the video might have positive externalities on less radical NGOs (*Assiettes Végétales* promotes plant-based meals at university canteens, and *Welfarm* supports animal farmers in improving their practice). In our setting, the video does not bring financial benefits to the NGO that published it (i.e., L214), which is presumably due to participants’ ignorance regarding its origin. However, the video seems to help the publishing NGO generate support for their demands, i.e., the ban on intensive farms. We discuss this question in more detail in the Discussion section below.

We also investigate whether the video worked at the extensive margin, i.e., whether it attracted new donors. While we cannot directly compare conditional donations due to composition effects (intensive margin), we compare the shares of participants who gave strictly positive amounts (Novid-Short vs Vid-Short). We observe similar results as above: *Assiettes Végétales*, *Welfarm*, and the petition against intensive farming increase their number of donors (+9.3pp, p $<0.001$; +8.3pp, p$<0.001$; and +10.4pp, p $<0.001$ respectively). The two NGOs that had no statistically significant increase in average donations (L214, SPA) also have the smallest change in the share of donors (+4pp, p = 0.112; and +0.5pp, p = 0.870 respectively). This suggests that the video also works at an extensive margin, attracting new donors.

Fourth, we do not see any difference in the moral concerns across treatment groups. Moral concerns for the animals are large in all groups, including in the baseline condition Novid-Short. On the 1-to-7 Likert scale, all items of the moral concerns for animals have average scores above 6 in all treatment conditions. Fifth, we observe some impact of the video on the pro-meat justifications (PMJ). We follow previous work^49,54^ and aggregate the PMJ items into a single PMJ score (average of the 10 items, Cronbach alpha: 0.91). Participants who saw the video display lower PMJ (Novid-Short: average: 0.571, CI: [0.558, 0.584], Vid-Short: 0.538 [0.525, 0.551], Wilcoxon $p<0.001$). However, the exploratory analysis suggests that this decrease in PMJ vanishes with the cool-off device (Novid-Short: 0.571 [0.558, 0.584], Vid-Long: 0.563 [0.550, 0.576], Wilcoxon $p=0.213$).

Sixth, we look at how participants who were exposed to the video perceived it. We analyze the answers given to the 1-to-7 Likert scales about the credibility of the video. On average, exposed participants tend to slightly disagree with the fact that the video is an exception and not representative of animal farming in France (average: 3.61 [3.53, 3.69]). They also reject the ideas that the video was recorded in another country (2.71 [2.63, 2.78]), that the video is fake (2.19 [2.12, 2.25]), that the information revealed in the video is not accurate (2.31 [2.25, 2.38]), that the information in the video is biased or distorted (2.37 [2.30, 2.44]), that the video is not believable (2.17 [2.10, 2.24]), or that the video is not realistic (2.20 [2.13, 2.27]). Overall, these results indicate that the participants have a fairly positive opinion of the credibility of the video.

Last, we look at the viral potential of this video. In the treatment groups with the video, we observe mixed positions about whether people would be willing to share the video on social media. When looking at the answers on the 1-to-7 Likert scale, we observe average answers close to the middle of the scale (3.91 [3.74, 4.07] in Vid-Short and 3.71 [3.54, 3.87] in Vid-Long). However, these averages hide a large heterogeneity in the willingness to share the video: about 31% of the participants in Vid-Short and 36% in Vid-Long report that it is ‘not at all likely’ that they would share the video on social media. On the contrary, 26% and 23% of the participants in these two groups say that it would be ‘very likely’ for them to share the video.

## Perception by activists

### Design

NGOs are faced with considerable resource constraints in their attempts to transform society. One key issue for activists is then to focus on the most-efficient strategies to achieve their desired change. However, activists may differ from the general population in a number of dimensions (e.g., knowledge, concern about the topic, demographic composition, and the type of networks they have), such that their perception of the effectiveness of their campaigns might be inaccurate. Regarding undercover videos, the expected total effect on engagement and the role of emotions in reactions may well be central to the allocation of resources between different campaigns.

We here propose to investigate whether activists have accurate perceptions of the total and mediated effects of videos. To do so, we recruited 154 activists from the NGO that produced the original video used in the experiment above. These activists were recruited via posts on the NGOs’ Facebook groups for activists, posted by members of the NGO themselves [See Note 9 in the Supplementary Materials]. The activist participants were blind to the study’s objective, and were only told that they would be asked about their opinions on a number of strategies implemented by French animal-advocacy NGOs. Our sample is 78% female, with an average age of 41, and has been active in the NGO under consideration for 4.4 years on average.

We designed a prediction study to assess the perception of activists regarding the role of the emotions produced by these videos [See Note 10 in the Supplementary Materials]. The text of the prediction study appears in the Supplementary Materials. In this prediction study, we ask activists to predict as accurately as possible the average donation levels and emotional scores that we obtain from the main experiment under the three conditions (Novid-Short, Vid-Short and Vid-Long). We first present activists with the design and population of the main experiment and ask a number of comprehension questions. Second, we ask them to watch the video included in the main study [See Note 11 in the Supplementary Materials]. Third, we ask participants their predictions about the average emotional state and donation levels produced in the main study. Note that activists are incentivized to give accurate predictions [See Note 12 in the Supplementary Materials].

The two main variables of interest are the emotional state and donation predictions. Regarding the emotional state, we detail to the activist how we constructed the aggregated emotional score: Participants had to report the intensity of several emotions they experienced, and we computed the sum of the reported scores for negative emotions minus the score. To simplify the prediction decision, we ask activists to assess the expected average emotional score on a normalized scale (slider task) taking values between 0% (very negative emotional state) and 100% (very positive emotional state). Regarding the donation prediction, we present to the activists the list of NGOs and petitions that were presented to the representative sample together we the donation decision they faced. Activists must report the average donation score (slider task) taking values from 0% (out of the 7 associations/petitions, the participant did not donate anything to any of the associations/petitions) and 100% (out of the 7 associations/petitions, the participant systematically donated all his/her money).

### Exploratory results

Data collection took place in January 2024 in accordance with GDPR regulation. Informed consent was obtained from all the study participants. In total, 50 activists completed the survey and gave their consent. While no outcome-neutral test was pre-registered regarding this part of the study, we underline that the limited number of observations urges for a prudent interpretation of the activists’ answers. On the other hand, let us also note that this type of audience is harder to reach than representative samples of the general population. Before discussing the results, we would like to stress three methodological points. First, interpreting the absolute emotional score, at least in the baseline group, makes little sense as it might be difficult for activists to predict absolute scores on such an unfamiliar scale. Second, we discussed for the main study differences in averages across groups (donations or emotions), and the mediated treatment effect is itself a ratio of differences in averages. However, the picture is different for the prediction study as activists must predict the reactions to each treatment. Because of the Simpson paradox^55^, note that the ratio of averages differs from the average of ratios. Last, because of the small number of observations, we opt for discussing the median view among activists rather than the average view to limit the influence of outliers^56^. As a result, we discuss below, when relevant, ratios of averages for the main study (between-subject design) and the median of ratios for the prediction study (within-subject design).

**Figure 9:**
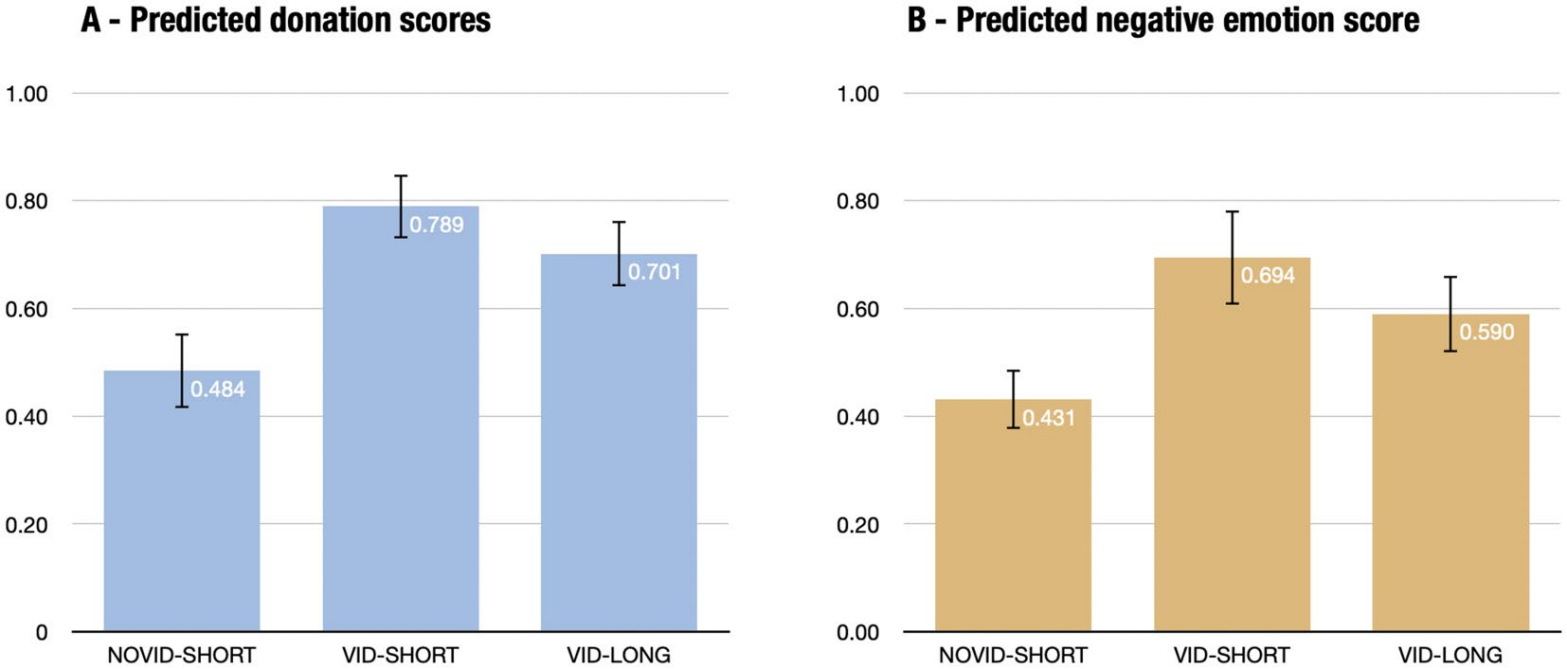
Expected average donations and emotional scores across treatments by activists. Bars display average scores per treatment. Spikes represent 95% confidence intervals.

The main results of the prediction study are displayed in Fig. 9. First, concerning donations, we observe that activists largely overestimate the altruistic actions of laypeople in the control group. The median expectation among activists is for participants in the main experiment to give on average 43.5% of their endowment while they only gave 16.4%. Second, while activists correctly anticipate an increase in donations after the video, they overestimate its size (both in absolute and relative values). The median expectation among activists is that the video increased donations by 28pp (+60.3%) while it increased average donations by 3.8pp (+23%) only. As far as emotions are concerned, activists correctly guess that participants experience more negative emotions after viewing the video, but they tend to slightly underestimate the size of the difference. The median expectation among activists is that negative emotions would increase by 35pp while they increased by 40.3pp. Because activists overestimate the negative emotional score in the control group, the median expectation among activists underestimates the relative difference in negative emotions (+85.5% vs. +156.8%).

Regarding the cool-off, activists correctly anticipate that the cool-off device mitigates the effect of the video on donations but they underestimate its effect on the emotional load. On the one hand, the median expectation among activists is that the cool-off period mitigates 36.5% of the effect of the video on donations, which is close to what we observe in the main experiment (28.9%). Note however that, in absolute value, the median expectation among activists regarding the decrease in donation is larger (−7pp) than what we observe (−1.1pp). On the other hand, the median expectation among activists is that 41.2% of the emotional effect of the video would be offset by the cool-off device against 83.9% in our data. Altogether, most of the activists anticipate a positive mediated treatment effect (76% of the activists’ sample).

## Discussion

Our experiment aimed at investigating the impact of undercover videos on people’s propensity to support social change. From our registered analyses, we first observe that these videos are successful in generating engagement. Watching the video about animal abuse in farms significantly increases donations in favor of animals. Second, our registered analysis fails to detect a direct role of emotions in this increase in engagement but, at the same time, also fails to reject the idea of an economically significant role of emotions. Indeed, we find a 17.5% chance that the mediated treatment effect is lower or equal to 0, but also a 10.8% chance that it is larger than 3pp (our registered threshold for economic significance). An exploratory analysis suggests that the mediated treatment effect would account, at the median, for 34.4% of the total treatment effect. However, the uncertainty of this estimate is large: the two-sided 95% confidence interval is equal to [0, 0.94]. Overall, the evidence is inconclusive on the mediated treatment effect, but, should there be a mediated treatment effect of emotions, the current balance of evidence tends to indicate that it would be of limited size.

As far as emotions are concerned, we observe that the video first generates a large negative emotional load. Exploratory analyses suggest that this negative shock is mostly due to an increase in sadness, anger, and disgust and a decrease in happiness. The video is much less likely to induce fear or surprise. While increases in donations tend to survive when the negative emotions vanish, we cannot exclude that they play a significant role in the video’s impact. The literature on emotions has shown that they can shape decisions via the content of the thought, the depth of the thought, and goal activation^16^. In other words, the negative emotional load might lead people to think about abuses in animal farming (content), to think more about it (depth), and to motivate them to act on this issue (goal activation). This is particularly true for anger which has been shown to stimulate motivation to fight injustice and which is here significantly affected by the video^7,19,57^. As discussed in the theoretical section, affects can also be informative about a situation: the negative emotions that a participant feels watching the video can contribute to their change of perception of the issue at stake. In addition, while we focused on primary emotions only, these can, in turn, generate longer-term and more elaborate emotions that can survive their disappearance, e.g., so-called secondary emotions^58^. Such secondary emotions, which include guilt and shame,^59,60^ were not captured in our study. They typically involve the self-concept^58^ and can persist even after the primary emotions have vanished. Another point of caution concerns the external validity of our definition of emotions which might not be universally applicable. For instance, recent work has suggested that individuals with limited exposure to Western concepts of emotion might perceive and classify facial expressions and emotional vocalizations differently compared to Western individuals^61–63^. Additionally, the decomposition of the emotional state into distinct emotions might also lack biological support, and they might result from non-distinct biological systems^64–66^.

Relatedly, the absence of statistical evidence supporting a causal role of felt emotions on subsequent behaviors in our study might well align with recent works in psychology on anticipated emotions. Several authors have indeed challenged the common assumption that emotions directly influence behaviors and have advanced the idea that people adapt their behavior ex-ante such as to avoid anticipated negative emotions. For example, some authors have argued that emotions function more as a feedback system rather than a direct cause of behavior, suggesting that behaviors are modified based on the learning and cognitive appraisal derived from emotional feedback^67^. Similarly, some other works have found that felt emotions do not consistently translate into behaviors, emphasizing the greater influence of anticipated emotions, such as shame or guilt, on behavior^68^.

Regarding donations, exploratory analysis suggests that less radical measures benefit the most from the videos (ban on intensive farms, development of plant-based menus in university canteens with *Asiettes Végétales*, support for farmers’ initiatives aimed at improving farm-animal welfare with *Welfarm*). Banning intensive animal farming is already a popular policy in France, where about 84% of the population supports a ban (IFOP, Jan. 2024). The video seems to stimulate this policy support by creating larger engagement. More moderate NGOs like Welfarm also benefit from the situation. Overall, it seems that participants do not want to stop meat consumption but support initiatives to improve the welfare of farmed animals. This result is of particular interest for the literature on the *radical flank effect*, which questions whether moderate groups benefit or suffer from the existence of more radical groups^49^. Evidence from donations suggests here a positive radical flank effect, as Welfarm, which is usually considered as more moderate than L214^69^, experiences an increase in donations. However, one important characteristic of our design is that participants ignore that the video was published by L214. In natural contexts, people are likely to know the identity of the NGO that published the video, which might change the donation patterns and might, in fine, mitigate the positive radical flank effect.

The credibility of these videos is often questioned by public officials or by the industry as a strategy to discredit its content. For instance, in 2020, a co-owner of a duck farm where L214 recorded and published footage of animal abuse declared that “the video is false, misleading and dishonest” [See Note 13 in the Supplementary Materials]. From a legal perspective, the NGO protects itself by having long unedited takes when recording on-site in which activists put various elements to prove the date of the recording (e.g., newspaper) and its location [See Note 14 in the Supplementary Materials]. The population seems to trust these videos: participants in our experiment show very little adherence to the idea that the video would be fake, distorted, or unbelievable. Among the usual arguments that we identified on this topic, participants show the highest adherence to the idea that the video is not representative of animal farming in the country. This could result from a sincere belief in the issue but could also be part of a strategy to avoid cognitive dissonance. Recently, we have seen L214 focusing most of its campaigns against specific brands (e.g., Burger King, Domino’s Pizza, LDC), which can contribute to fighting against the dilution of responsibility resulting from the ‘non-representativeness’ argument.

Although the undercover videos seem to achieve their objective of increased engagement, we must note however that our experimental setting is highly controlled. Some factors might substantially limit the impact of the video outside of this setting. For instance, participants might self-select, and only sympathizers could view the video. While the publication of the video might help sustain engagement in the long run, we might not have the extensive margin effect that our exploratory analyses underlined. In other words, it might fail to bring new people to act for the animals. Another example: When a new video is released, the industry and the government might also react publicly, as we have discussed in the previous paragraph. This might give people counter-arguments to discount the message of the NGO. These arguments can be about the farm itself (e.g., the public veterinary services controlled the farm and did not report any transgression [See Note 15 in the Supplementary Materials] or about other dimensions (e.g., the farmer is in economic difficulty because of the publication of the NGO [See Note 16 in the Supplementary Materials]. Overall, our study might therefore overestimate the effect of the videos in uncontrolled contexts, and future research is needed in this direction.

From a methodological perspective, our experiment aimed to identify the effect of videos on donations and emotions. Following the recent advances in mediation analysis, we developed an empirical strategy with two treatment variations to causally estimate the mediated treatment effect of emotions. We believe that our paper makes here two noticeable contributions to the literature. First, the model that we developed in “Analysis plan” can easily be used in other causal mediation analyses. This model is relatively parsimonious in terms of statistical assumptions. It relies indeed on two major assumptions: the absence of defiers (which we justify in the model as the fact that no one would feel better by viewing the video), and the assumption that the group of compliers who react to the cool-off (i.e., their negative emotions vanish) and the group of compliers who do not react to the cool-off (i.e., their negative emotions persist) react in the same way to the video treatment (i.e., on expectation, they similarly increase their donations when exposed to the video). . The model identifies a linear mediated treatment effect as a linear regression would capture. Second, we contribute to the literature that looks at the impact of primary emotions by showing that a five-minute cool-off might be enough to mitigate the main treatment effect on the mediator (here: how the five-minute transport survey mitigates most of the impact of the undercover video showing animal abuse on the emotional state). In our experiment, the cool-off did not impact the outcome variable (donations), but future works that would use our method can also use our alternative estimator presented in the Supplementary Materials, should the cool-off have an effect per se on the outcome variable (i.e., in the absence of the main treatment).

Another important methodological question in this type of experimental setting is the definition of the benchmark situation with which we compare the variables of interest. In the context of emotions, defining a *neutral affect* is difficult, if not impossible, as there is no neutral situation in terms of emotions^70^. In our case, we compared participants’ decisions and emotions to a baseline situation where they were not exposed to any video. This aims to reflect real-life situations where, for instance, participants are asked to donate to a charity on a social media platform, and some participants were previously exposed to an undercover video (by a friend liking or sharing the video for instance). Alternative experimental designs could display a neutral video in the control group such as to ensure that there is no video effect per se on the participants. The main drawback is that there is no such thing as a completely neutral video (i.e., videos that induce no primary emotions could induce boredom), and the empirical results could depend on the specific video chosen for the baseline.

Last but not least, our prediction survey brings new knowledge about how activists see the population and how they expect them to react. First, our exploratory analysis suggests that activists overestimate the general population’s altruistic behaviors. Activists overestimate indeed donations in the control group and expect a larger increase in engagement after watching the video. We postulate that activists might have a distorted optimistic view of general altruism towards animals, which could result from several behavioral factors (e.g., false consensus effect^71^, majority illusion^72^, echo chamber^73^, projection bias^74^). This distorted perception of the general population’s concerns and behaviors regarding the cause an NGO is willing to defend could, in the long run, threaten the effectiveness of its campaigns. Second, activists in our survey correctly anticipate that the video generates negative emotions among participants in our main experiment. Nevertheless, they expect the emotions to last longer than what we observe. This overestimation aligns with research on affective forecasting^75–77^, which refers to the process by which people predict their future emotional reactions to events. Overall, this research has shown that people often overestimate the impact and duration of their emotional reactions. This may explain what we observe here for the activists’ predictions about the duration of the negative emotions among others resulting from watching a video. Here also, it is possible that activists are more sensitive to animal abuse and, because they think that they would be affected longer by the video, they expect the general population to do so. Overall, this leads activists to overestimate the role of emotions in the increased engagement in favor of animals induced by the video. However, these results should be interpreted with caution given the small number of observations and given that they are only exploratory analyses.

## Supplementary Information

Supplementary Information.

## Data Availability

The data and codes to replicate the paper are available here: https://zenodo.org/doi/10.5281/zenodo.10777689.
