# Supplementary material for: Impact of NGOs’ undercover videos on citizens’ emotions and pro-social behaviors
Source: Sci Rep. 2024 Sep 4;14:20584. doi: 10.1038/s41598-024-68335-5 (PMC11374992; doi:10.1038/s41598-024-68335-5)
Supplement: Supplementary file 1 — Supplementary Information. [file 41598_2024_68335_MOESM1_ESM.pdf]

Registered Report - Stage 2:

Impact of NGOs' Undercover Videos on Citizens' Emotions and Pro-Social Behaviors

Romain Espinosa, Sylvie Borau, Nicolas Treich

August 16, 2024

Supplementary Notes:

- Note 1: Sources: NGO's website for Mercy for the Animals, GuideStar's data for Animal Equality.
- Note 2: Source: L214's website
- Note 3: Authors' own translation. Original source: `https://web.archive.org/web/20230213124740/https://www.radiofrance.fr/franceinter/podcasts/magma-le-mag-de-la-matinale/le-mag-de-la-matinale-du-lundi-29-aout-2022-5984035`.
- Note 4: The construction of the negative-emotion score was supported by a Principal Component Analysis. The first dimension of a PCA on emotions in the final sample explains 56.3% of the variance in emotions (65.2% in the pilot sample). It correlates positively with all emotions but with happiness (negative correlation). All correlation coefficients are larger than 0.60 in absolute value in both the pilot and final samples except for surprise in the final sample (0.40).
- Note 5: In the pilot study, we retained participants who did not report any sound as this question was not mandatory when we launched the survey. In the final experiment, we dropped all participants who did not provide the correct answer to the sound question.
- Note 6: The parameter δ is equivalent to an interaction effect between the video and the long treatments in a standard linear regression.
- Note 7: The Stage-1 manuscript did not contain any rationale about the 3 percentage-point cut-off which was defined arbitrarily. A referee asked during the Stage-2 review for a rationale. We provide ex-post the following information: a 3 percentage-point increase in donations corresponds to a Cohen's d of 0.18 in the pilot data using the Novid-Short condition as baseline. This effect size is usually considered as small (i.e., $d < 0.2$[51]).
- Note 8: The number of participants slightly exceeds the pre-registered number of participants (N=3,200) because the survey company invited more respondents than needed to account for potential drop-outs.
- Note 9: The participants were recruited in two waves. A first group of participants were recruited in June 2022, and a second group in September 2023. In September 2023, participants who registered in June 2022 were asked to confirm their willingness to participate in the study. The final sample of 154 participants comprises the participants recruited in 2022 who confirmed their participation and the participants recruited in 2023.
- Note 10: This study received the approval of the ethics committee of the Institute for Advanced Studies in Toulouse in February 2023 (IRB approval number: reference 2023-02-003).

- Note 11: We decided to ask activists to watch the video themselves before reporting their beliefs about what participants in the group who had seen the video would do, as well as what participants who had not seen the video would do for reasons of external validity: when activists decide whether to release an undercover video they know what the video shows and it is with this knowledge that they predict the individuals' reactions to it. Note that we did not include Novid-Long as this treatment was not relevant to the prediction study.
- Note 12: We told activists that one of them would be randomly selected at the end of the prediction study and that one of her predictions would be randomly selected. If this prediction was sufficiently close to the correct answer (within a 5 percentage-point interval around the correct answer), we would donate 100 Euros to the NGO under consideration.
- Note 13: Link: https://web.archive.org/web/20240120074009/https://www.lefigaro.fr/flash-eco/elevage-insalubre-de-canards-la-video-de-l214-est-mensongere-selon-la-coproprietaire-20200820
- Note 14: Example: https://www.youtube.com/watch?v=IhsZcjJ7PaE
- Note 15: Link: https://web.archive.org/web/20240227055314/https://www.francetvinfo.fr/animaux/bien-etre-animal/vendee-les-autorites-n-ont-trouve-aucune-non-conformite-dans-l-elevage-mis-en-cause-par-l214_2220053.html
- Note 16: Link: https://web.archive.org/web/20240227055546/https://www.lepoint.fr/societe/cible-par-une-video-de-l214-un-elevage-en-liquidation-judiciaire-11-07-2019-2324110_23.php

Supplementary Materials 1: Experimental design

Experiment

Intro
Dear Participant,
In this study, we are going to ask you questions about your diet and food consumption as well as your beliefs, attitudes, and opinions on current topics, and some demographic questions.
The study should last about 20 minutes.
The information provided in this questionnaire will only be used for academic/scientific purposes (no commercial purposes).
You will be compensated in points, as indicated on the previous page.

Confidentiality, Disclosure of Information, and Declaration of Voluntary Participation
You will not be asked for your name or any other information that could be used to identify you. The information you provide will be stored anonymously on the Qualtrics account for two weeks and can never be linked to you, your real name, or your IP address. Anonymous data will initially be accessible only to the team of researchers involved in this project, then the anonymized data will be stored indefinitely on a data-sharing account for the purpose of replication and to promote reproducible science. Participation is completely voluntary. You can end your participation at any time.
If you have any questions about this study, you can contact Sylvie Borau: s.borau@tbs-education.fr
We thank you for your participation!

Gender What is your gender?

○ Man (1)

○ Woman (2)

○ I don't identify as a man or a woman (3)

Captcha Please click on the link below.

Risks Disclaimer and Consent:

In the following questionnaire, you may be exposed to explicit images that may elicit unpleasant levels of emotion. These feelings are usually temporary.

○ I am aware of the explicit content of this study. (4)

Consent I have read this form and am aware that I am being asked to participate in a study that may contain explicit images.
I voluntarily agree to participate in this study. I can end my participation at any time.

- ○ I agree to participate in this study (2)
- ○ I do NOT agree to participate in this study (4)

Does not consent
As you do not wish to participate in this study, the questionnaire ends here. Thank you for your participation.

Randomization
The following two questions are randomized in the questionnaire. Half of the sample is exposed to these two questions at the beginning of the questionnaire; the other half at the end of the questionnaire.

Food consumption Please indicate how often you consume the following items:

	Never (1)	A few times a year (2)	A few times a month (3)	A few times a week (4)	Almost at every meal (5)
Red meat (1)	○	○	○	○	○
White meat (2)	○	○	○	○	○
Fish (3)	○	○	○	○	○
Eggs (4)	○	○	○	○	○
Dairy products (5)	○	○	○	○	○
Vegetables (6)	○	○	○	○	○
Pitch/ pulses (7)	○	○	○	○	○
Fruits (8)	○	○	○	○	○
Starchy foods (9)	○	○	○	○	○

Diet identity And overall, do you identify the most as a...

- ○ Vegetarian (1)
- ○ Vegan (2)
- ○ Omnivore (3)
- ○ Carnivore (4)
- ○ Flexitarian (5)

Sound In the rest of the questionnaire, you may be exposed to a video. That is why we would like to make sure you can listen to sounds on your device

Below, you will be asked to listen to a sound and then indicate the sound you heard.

If you do not answer the question correctly, you will not be able to continue this study.

Sound test Please listen to this sound clip and indicate which sound it is by selecting the correct answer below.

- ○ Honking car alarm (1)
- ○ Crickets (2)
- ○ A cat meowing (3)
- ○ A person whistling (4)
- ○ A dog barking (5)

Error sound You have not selected the correct answer. As you cannot play sound on your device, the questionnaire ends here. Thank you for your participation.

Intro video On the next page, you will be exposed to a video showing animal intensive farming in France.
This investigation took place over several months and used hidden cameras.
Please watch this video carefully. Note that the video is only one minute and 20 seconds long.

Video Please watch the video by clicking directly on the video. You don't need to go to Vimeo to watch it.

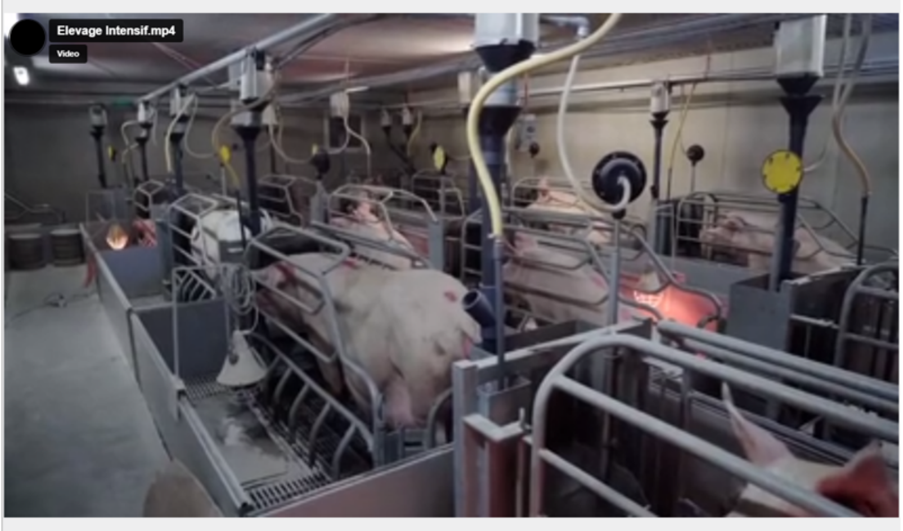

[In the LONG treatment, the questionnaire about the transportation mode is displayed here.]

Intro emotions

Now we would like to know how you feel right now.

We would like you to look at some pictures of faces and tell us which picture of faces best matches with how you feel right now.

Emotions Choice Please click on the set of faces that best matches your emotions.

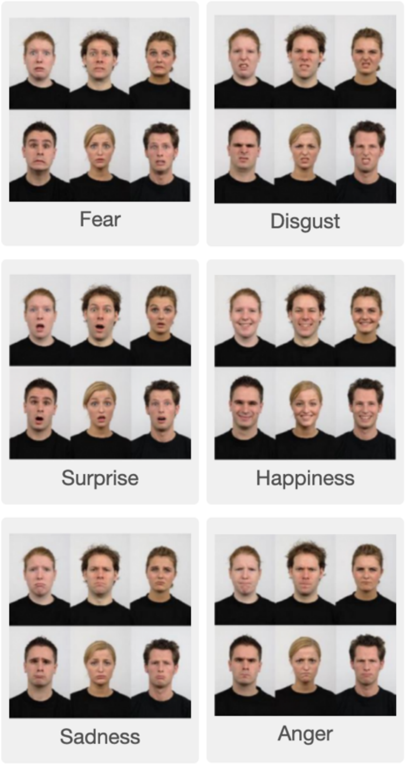

- ◯ (1)
- ◯ (2)
- ◯ (3)
- ◯ (4)
- ◯ (5)
- ◯ (6)

IntroEm2 Now you will see each set of faces separately, and you will rate how well each set of faces matches how you feel right now.

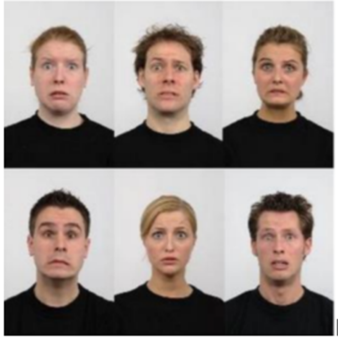
Fear

Please indicate how strongly you feel this emotion.

- ◯ 1. Strongly disagree (1)
- ◯ 2 (2)
- ◯ 3 (3)
- ◯ 4 (4)
- ◯ 5 (5)
- ◯ 6 (6)
- ◯ 7. Strongly agree (7)

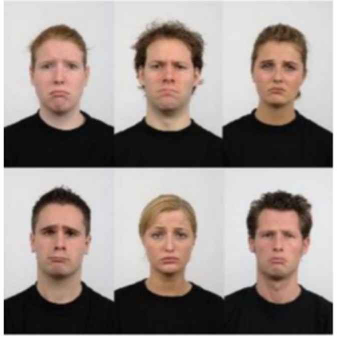

Sadness

Please indicate how strongly you feel this emotion.

- ○ 1. Strongly disagree (1)
- ○ 2 (2)
- ○ 3 (3)
- ○ 4 (4)
- ○ 5 (5)
- ○ 6 (6)
- ○ 7. Strongly agree (7)

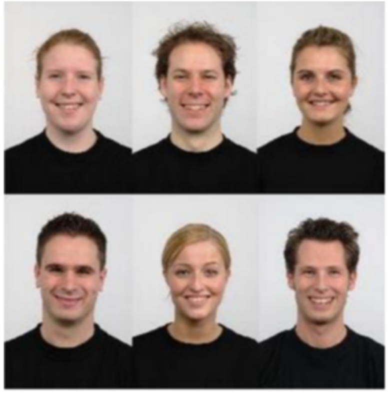

Happiness

Please indicate how strongly you feel this emotion.

- 1. Strongly disagree (1)
- 2 (2)
- 3 (3)
- 4 (4)
- 5 (5)
- 6 (6)
- 7. Strongly agree (7)

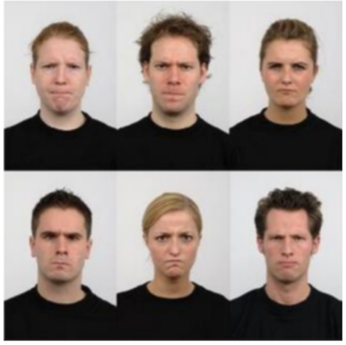

Anger

Please indicate how strongly you feel this emotion.

- 1. Strongly disagree (1)
- 2 (2)
- 3 (3)
- 4 (4)
- 5 (5)
- 6 (6)
- 7. Strongly agree (7)

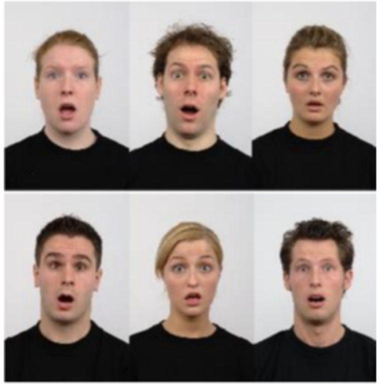

Surprise

Please indicate how strongly you feel this emotion.

- 1. Strongly disagree (1)
- 2 (2)
- 3 (3)
- 4 (4)
- 5 (5)
- 6 (6)
- 7. Strongly agree (7)

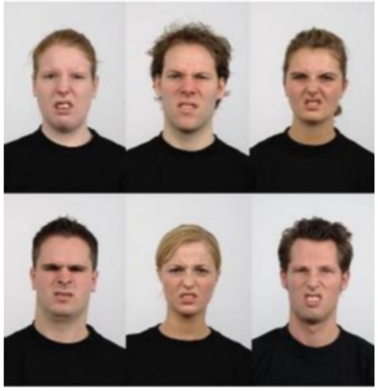

Disgust

Please indicate how strongly you feel this emotion.

- 1. Strongly disagree (1)
- 2 (2)
- 3 (3)
- 4 (4)
- 5 (5)
- 6 (6)
- 7. Strongly agree (7)

Charity

In addition to the points you will receive for taking part in this survey, we will give you an additional 1 Euro which will be converted into points.

You have the option of keeping this money (the points will be credited to your account) or donating some or all of this extra money.

We present below a list of associations and petitions that you can support by making donations. For information, a donation for a petition will help increase its visibility on social networks for example.

Note that at the end of the survey, only one of your decisions will be implemented depending on the association or petition that we selected upstream.

Please indicate for the 7 associations or petitions below if you wish to donate money (knowing

that only one association or petition will receive the donation, the remaining 6 being hypothetical). Note that all the actions and petitions mentioned actually exist.

<u>Lists</u> List of proposed French associations:

- Vegetable Plates: An association that seeks to put more vegetarian and vegan meals in university canteens.

- L214: An animal protection association that denounces the most cruel practices of the livestock industry and seeks to develop diets without animal products

- SPA: An association that works to protect animals, mainly domestic animals (cats, dogs), for example by fighting against abandonment and encouraging adoption.

- Welfarm: An association that supports the initiatives of breeders and the agri-food industry that contribute to improving the well-being of animals on farms

List of petitions:

- Against intensive farming: A petition to ban intensive farming in France

- For vegetarian meals: A petition to have at least one vegetarian meal per day in all public canteens in France

- To protect activists: A petition to protect activists in France who record videos showing the mistreatment of animals in intensive farming

---

Donation associations (cents):

0 10 20 30 40 50 60 70 80 90 100

SPA

Wellfarm

Assiettes végétales

L214

Donation petitions (cents):

0 10 20 30 40 50 60 70 80 90 100

Against intensive farming

For vegetarian meals

To protect activists

Confirm I confirm the amounts indicated above: a line will be selected to be set up. The amount I indicated will be subtracted from the additional 50 cent bonus I will receive.

- I confirm (1)

Moral concern animal Please indicate to what extent you agree or disagree with the following statements about animals:

	1. Totally disagree (1)	2 (2)	3 (3)	4 (4)	5 (5)	6 (6)	7. Totally agree (7)
Animals deserve to be protected from harm (1)	o	o	o	o	o	o	o
Animals deserve to be treated with care and compassion. (2)	o	o	o	o	o	o	o
I have sympathy for animals (3)	o	o	o	o	o	o	o
I have respect for animals (4)	o	o	o	o	o	o	o
Harming animals is morally wrong (5)	o	o	o	o	o	o	o

Promeat Please indicate to what extent you agree with the following statements.

	1. Totally disagree (1)	2 (2)	3 (3)	4 (4)	5 (5)	6 (6)	7. Totally agree (7)
It is acceptable to eat meat because the animals killed for our consumption do not really suffer. (2)	○	○	○	○	○	○	○
It is acceptable to eat meat because the animals killed for our consumption have lower intellectual capacities than humans. (7)	○	○	○	○	○	○	○
It is acceptable to eat certain animals because they are bred for that purpose. (9)	○	○	○	○	○	○	○
God created animals for us to eat (10)	○	○	○	○	○	○	○
Eating meat is healthy (11)	○	○	○	○	○	○	○
It is natural to eat meat, it is written in our genes. (12)	○	○	○	○	○	○	○
It's normal to eat meat (13)	○	○	○	○	○	○	○
I like meat too much to stop eating it. (14)	○	○	○	○	○	○	○
Eating meat is necessary for good health (15)	○	○	○	○	○	○	○
Eating meat may be bad for the environment, but no more so than eating vegetables or cereals. (16)	○	○	○	○	○	○	○

Morally wrong
How **morally wrong do** you think the animal rearing conditions shown in this video are.

Please rate how morally wrong these conditions are, using the scale below, where 0 is "not at all morally wrong" and 100 is "extremely morally wrong."

	**Not morally wrong**	**Morally wrong**
	0 10 20 30 40 50 60 70 80 90 100	
These rearing conditions are... ()		

Sharing If you were to see the above video on Twitter or Facebook, how likely would you be to share it?

- ○ 1. Not at all likely (1)
- ○ 2 (2)
- ○ 3 (3)
- ○ 4 (4)
- ○ 5 (5)
- ○ 6 (6)
- ○ 7. Very Likely (7)

Truth To what extent do you agree with the following statements?

	Completely disagree (1)	Strongly disagree (2)	Somewhat disagree (3)	Neither agree nor disagree (4)	Somewhat agree (5)	Strongly agree (6)	Completely agree (7)
This video is not representative of animal framing in France, it is rather an exception (1)	o	o	o	o	o	o	o
This video was not recorded in France but in another country. (2)	o	o	o	o	o	o	o
This video is fake (militant activists made it up). (3)	o	o	o	o	o	o	o
The information revealed in this video is not accurate (4)	o	o	o	o	o	o	o
The information revealed in this video is distorted and biased. (5)	o	o	o	o	o	o	o
This video is not believable (6)	o	o	o	o	o	o	o
This video is not realistic (7)	o	o	o	o	o	o	o

Eat Meat -motivation And to what extent do you agree with the following statement?

" Right now, I'm motivated to eat meat "

	Not agree at all										Totally agree
	0	1	2	3	4	5	6	7	8	9	10
Motivated to eat meat ()											

Check1 What is the main subject of this study?

○ Diet (10)

○ Artificial intelligence (11)

○ Obesity around the world (17)

○ Overpopulation in the world (18)

Check2 What did you see in the video you were exposed to? (several possible responses)

☐ chickens that got beaten up (1)

☐ animals helping humans (3)

☐ men and women eating beef (4)

☐ humans killing cows (5)

☐ pigs being mistreated (2)

Seen video - And have you seen this particular video before?

- ○ Yes (1)
- ○ No (2)

Seen this type of video - And have you seen a similar video recently about farm animal abuse?

- ○ Yes (1)
- ○ No (2)

Check 3 What is 12 + 40?

- ☐ 12 (1)
- ☐ 21 (3)
- ☐ 32 (4)
- ☐ 41 (5)
- ☐ 52 (2)
- ☐ 63 (8)

Age How old are you ?

◯ 18 (3)

◯ 75 or more (60)

Marital status What is your current marital status?

◯ Single (1)

◯ In a relationship, but not married (2)

◯ Married (3)

Children Do you have any children?

◯ Yes (1)

◯ No (2)

Political ideology Overall, what would be the best description of your political views?

	Far left					Center					Far right
	0	1	2	3	4	5	6	7	8	9	10
1 ()											

Religion Do you believe in God ?

- ◯ 1. No, not at all (1)
- ◯ 2 (2)
- ◯ 3 (3)
- ◯ 4 (4)
- ◯ 5 (5)
- ◯ 6 (6)
- ◯ 7. Yes, a lot (7)

Debrief Thank you for your participation in this study!

The objective of this questionnaire was to better understand the influence of videos denouncing animal abuse in intensive farms on the beliefs and behavior of consumers in terms of meat consumption. This is an online experiment: half of the participants were exposed to a video showing intensive farm animal abuse, and the other half were not exposed to any video. The same questions were then asked to all participants. The ultimate goal is to explore the role of emotions in consumer reactions to animal abuse and meat consumption.

Here are some online resources if you want to learn more about farm animal abuse and advocacy:

Factory farms are multiplying all over the world:
https://www.la-croix.com/Economie/France/Greenpeace-dresse-carte-fermes-usines-France-2018-11-26-1200985667

The defense of farm animals claimed by moderate associations:
https://www.lepoint.fr/societe/la-defense-des- Animaux-de-ferme-revendiquee-par-des-associations-moderees-12-10- 2018-2262473_23.php

The peaceful retirement of farm animals that have survived slaughterhouses or mistreatment:
https://www.lemonde.fr/m-perso/article/2021/02/26/rescapes-des-abattoirs-de-l-industrie-ou-de-la-maltraitement-ces- Animaux-de-ferme- flow-a-peaceful-retreat_6071343_4497916.html

---

Figure SM1: Design of the experiment

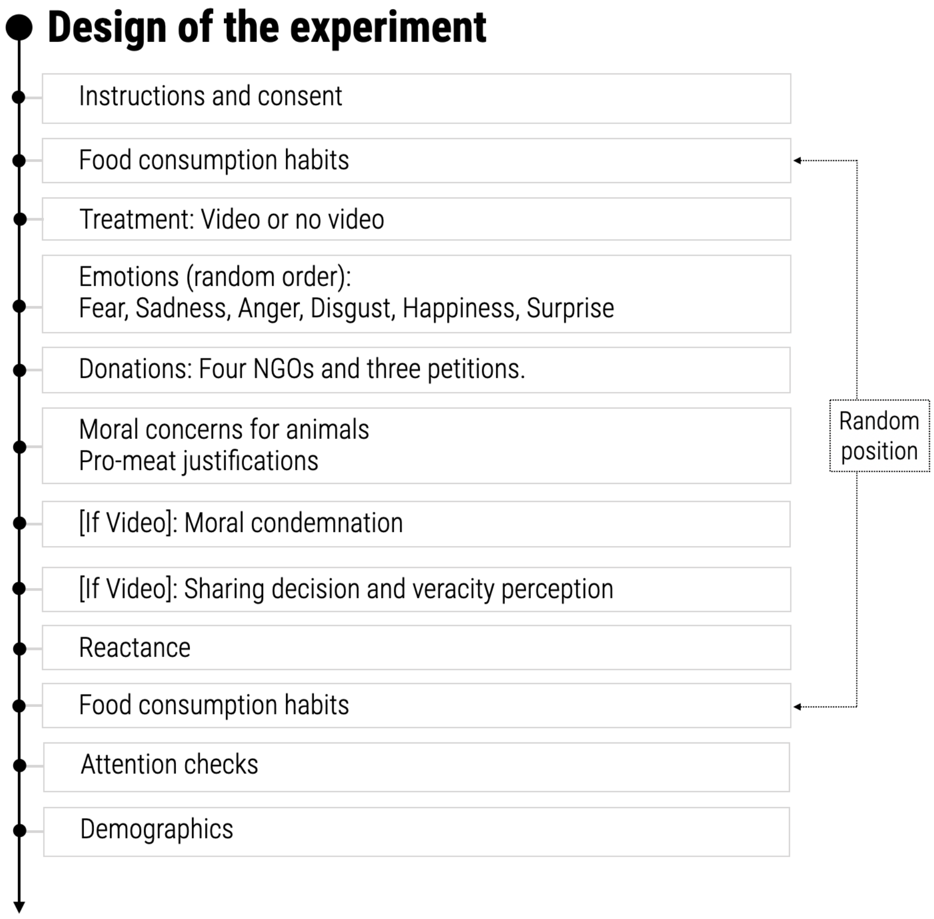

Supplementary Materials 2: Alternative estimator for the mediated treatment effect

Based on the pilot data, we assume in the Stage-1 manuscript that there is no effect of the Long treatment *per se* (i.e., no change in donations associated with having the long questionnaire in the absence of the video). We find some empirical support for this assumption as we fail to reject the null hypotheses of equality of donations between Novid-Short and Novid-Long in the pilot sample (Wilcoxon rank-sum test: Novid-Short vs. Novid-Long: $p > 0.477$ for all of the single-donation decisions). However, we cannot exclude that, in the main experiment, this assumption will not hold. In the Stage-1 manuscript, we commit to running an outcome-neutral test to test this assumption on the final sample data. If we reject the null hypothesis of equality of donations on the aggregate donation score (Wilcoxon rank-sum test: Novid-Short vs. Novid-Long) and if the difference is larger than 2 percentage points (i.e., economically significant difference), we commit to using the following alternative estimator for the mediated treatment effect instead of the estimator presented in the main manuscript.

We now relax the assumption that the Long version of the questionnaire has no effect on donations in the absence of video. It follows that our cool-off device might have two effects on donations: a direct effect on donations and an indirect effect through the mitigation of negative emotions. The indirect effect occurs only in the Vid-Long treatment, while the direct effect occurs in both Vid-Long and Novid-Long treatments.

Let us start by introducing a change in notation. We now allow the potential outcome to depend on the duration of the questionnaire. We write the potential outcome $Y_i^{d,m,l}$ where $l = 1$ if the participant has faced the long questionnaire and $l = 0$ if she has faced the short questionnaire. We assume that the direct effect of the long questionnaire affects on average the always-takers, the never-takers, and the compliers in a similar way. Let us note μ this direct effect. Because the long questionnaire has only a direct effect for participants who are not exposed to the video (i.e., no emotional mediation), we have: $\mu = \mathbb{E}[Y_i|D_i = 0, C_i = 1, T_i] - \mathbb{E}[Y_i|D_i = 0, C_i = 0, T_i]$.

Next, we define a new mediated treatment effect Δ'_M which is equal to:

$$\begin{aligned}\Delta'_M &= \mathbb{E}[Y_i^{1,M_i^1,0} - Y_i^{1,M_i^0,0}|D_i = 1] \\ &= p_c\mathbb{E}[Y_i^{1,M_i^1,0} - Y_i^{1,M_i^0,0}|D_i = 1, T_i = c] \qquad (10)\end{aligned}$$

We now define a parameter δ':

$$\begin{aligned}\delta' &= \mathbb{E}[Y_i|D_i = 1, C_i = 0] - \mathbb{E}[Y_i|D_i = 1, C_i = 1] - (\mathbb{E}[Y_i|D_i = 0, C_i = 0] - \mathbb{E}[Y_i|D_i = 0, C_i = 1]) \\ &= \mathbb{E}[Y_i|D_i = 1, C_i = 0] - \mathbb{E}[Y_i|D_i = 1, C_i = 1] + \mu \qquad (11)\end{aligned}$$

It follows:

$$\begin{aligned}\delta' =& p_a \times (\mathbb{E}[Y_i^{110}|D_i = 1, C_i = 0, T_i = a] - \mathbb{E}[Y_i^{111}|D_i = 1, C_i = 1, T_i = a]) \\ &+ p_n \times (\mathbb{E}[Y_i^{100}|D_i = 1, C_i = 0, T_i = n] - \mathbb{E}[Y_i^{101}|D_i = 1, C_i = 1, T_i = n]) \\ &+ p_c \times \{\mathbb{E}[Y_i^{110}|D_i = 1, C_i = 0, T_i = c] \\ &- (1-q)\mathbb{E}[Y_i^{111}|D_i = 1, C_i = 1, T_i = c] - q\mathbb{E}[Y_i^{101}|D_i = 1, C_i = 1, T_i = c]\} \\ &+ \mu\end{aligned} \tag{12}$$

Using the fact that $\mathbb{E}[Y_i^{d,M,1}|D_i, C_i, T_i] = \mathbb{E}[Y_i^{d,M,0}|D_i, C_i, T_i] + \mu$ (i.e., for a fixed level of the mediator, the change in average donation due to the direct effect of the long questionnaire is equal to μ), we obtain:

$$\begin{aligned}\delta' =& p_a \times (\mathbb{E}[Y_i^{110}|D_i = 1, C_i = 0, T_i = a] - \mathbb{E}[Y_i^{110}|D_i = 1, C_i = 1, T_i = a] - \mu) \\ &+ p_n \times (\mathbb{E}[Y_i^{100}|D_i = 1, C_i = 0, T_i = n] - \mathbb{E}[Y_i^{100}|D_i = 1, C_i = 1, T_i = n] - \mu) \\ &+ p_c \times \{\mathbb{E}[Y_i^{110}|D_i = 1, C_i = 0, T_i = c] \\ &- (1-q)\mathbb{E}[Y_i^{110}|D_i = 1, C_i = 1, T_i = c] - q\mathbb{E}[Y_i^{100}|D_i = 1, C_i = 1, T_i = c] - \mu\} \\ &+ \mu\end{aligned} \tag{13}$$

Simplifying the equation, we get:

$$\begin{aligned}\delta' =& -\mu(p_a + p_n + p_c) + p_c \times q \times \mathbb{E}[Y_i^{110} - Y_i^{100}|D_i = 1, T_i = c] + \mu \\ =& p_c \times q \times \mathbb{E}[Y_i^{110} - Y_i^{100}|D_i = 1, T_i = c]\end{aligned} \tag{14}$$

The new mediated treatment effect is then equal to:

$$\Delta'_M = \frac{\delta'}{q} \tag{15}$$

Empirically, the parameter δ' is estimated by a difference-in-difference in the average donation levels:

$$\hat{\delta}' = (\overline{Y}_{\text{VS}} - \overline{Y}_{\text{VL}}) - (\overline{Y}_{\text{NS}} - \overline{Y}_{\text{NL}}) \tag{16}$$

Supplementary Materials 3: Estimation of sample size based on precision analysis

```r
#Import libraries
library(doParallel)
library(MASS)

#Set parameters
B=1000 #Bootstrap for estimating SE
S=1000 #Bootstrap for estimating precision power
N_vec=c(700,800) #Vector of sample sizes. Observations per treatment

#Set working Directory
#setwd("")

#Set seed
set.seed(123)

#Import data
mydata=readRDS("Data/treatedData.RDS")

#Function to estimate SE
estimateSE=function(B_funct,data_funct){

  #Create subdata for bootstrapping
  data_SHORT_NOVID_funct=data_funct[data_funct$treatmentVideo==0 & data_funct$treatmentLongSurvey==0,]
  data_SHORT_VID_funct=data_funct[data_funct$treatmentVideo==1 & data_funct$treatmentLongSurvey==0,]
  data_LONG_VID_funct=data_funct[data_funct$treatmentVideo==1 & data_funct$treatmentLongSurvey==1,]

  #Store the results
  storeDelta_M_funct=rep(NA,B_funct)

  #Loop to estimate SEs
  for(b_funct in 1:B_funct){

    #Draw data with replacement
    bootdata_SHORT_NOVID_funct=data_SHORT_NOVID_funct[sample(nrow(data_SHORT_NOVID_funct), nrow(data_SHORT_NOVID_funct), replace=TRUE), ]
    bootdata_SHORT_VID_funct=data_SHORT_VID_funct[sample(nrow(data_SHORT_VID_funct), nrow(data_SHORT_VID_funct), replace=TRUE), ]
    bootdata_LONG_VID_funct=data_LONG_VID_funct[sample(nrow(data_LONG_VID_funct), nrow(data_LONG_VID_funct), replace=TRUE), ]
    databoot_funct=rbind(bootdata_SHORT_NOVID_funct,bootdata_SHORT_VID_funct, bootdata_LONG_VID_funct)

    #Compute the mediated treatment effect
    delta_boot_funct=mean(databoot_funct[databoot_funct$treatmentVideo==1 & databoot_funct$treatmentLongSurvey==0,]$proAnimals)-mean(databoot_funct[databoot_funct$treatmentVideo==1 & databoot_funct$treatmentLongSurvey==1, ]$proAnimals)
    q_boot_funct=(mean(databoot_funct[databoot_funct$treatmentVideo==1 & databoot_funct$treatmentLongSurvey==0,]$emotionsAverage)-
                  mean(databoot_funct[databoot_funct$treatmentVideo==1 & databoot_funct$treatmentLongSurvey==1,]$emotionsAverage))/
      (mean(databoot_funct[databoot_funct$treatmentVideo==1 &
```

```
databoot_funct$treatmentLongSurvey==0,]$emotionsAverage)-
        mean(databoot_funct[databoot_funct$treatmentVideo==0 & databoot_funct$treatmentLongSurvey==0,]$emotionsAverage))
    Delta_M_boot_funct=delta_boot_funct/q_boot_funct

    #Store the result
    storeDelta_M_funct[b_funct]=Delta_M_boot_funct
  }

  return(sd(storeDelta_M_funct))

}

#Test the function
estimateSE(B_funct=B,data_funct=mydata)

#Function that estimate precision
estimatePrecision=function(N_funct,S_funct,B_funct,data_funct){

  #Create subdata for bootstrapping
  data_SHORT_NOVID_funct=data_funct[data_funct$treatmentVideo==0 & data_funct$treatmentLongSurvey==0,]
  data_SHORT_VID_funct=data_funct[data_funct$treatmentVideo==1 & data_funct$treatmentLongSurvey==0,]
  data_LONG_VID_funct=data_funct[data_funct$treatmentVideo==1 & data_funct$treatmentLongSurvey==1,]

  #Store the results
  storeSE_funct=rep(NA,S_funct)

  #Simulate data
  for(s_funct in 1:S_funct){

    #Draw data with replacement
    bootdata_SHORT_NOVID_funct=data_SHORT_NOVID_funct[sample(nrow(data_SHORT_NOVID_funct), N_funct, replace=TRUE), ]
    bootdata_SHORT_VID_funct=data_SHORT_VID_funct[sample(nrow(data_SHORT_VID_funct), N_funct, replace=TRUE), ]
    bootdata_LONG_VID_funct=data_LONG_VID_funct[sample(nrow(data_LONG_VID_funct), N_funct, replace=TRUE), ]
    databoot_funct=rbind(bootdata_SHORT_NOVID_funct,bootdata_SHORT_VID_funct, bootdata_LONG_VID_funct)

    storeSE_funct[s_funct]=estimateSE(B_funct=B_funct,data_funct=databoot_funct)
    #print(storeSE_funct[s_funct])
  }

  return(mean(storeSE_funct, na.rm=TRUE))
}

#Test function
estimatePrecision(N_funct=100,S_funct=10,B_funct=50,data_funct=mydata)

#Function for power analysis
#B: number of random draws for the bootstrap Standard Errors
#C: number of random draws for the power analysis
estimatePrecisionParallel=function(N_funct,S_funct,B_funct,data_funct){

  #Create subdata for bootstrapping
```

```
  data_SHORT_NOVID_funct=data_funct[data_funct$treatmentVideo==0 & data_funct$treatmentLongSurvey==0,]
  data_SHORT_VID_funct=data_funct[data_funct$treatmentVideo==1 & data_funct$treatmentLongSurvey==0,]
  data_LONG_VID_funct=data_funct[data_funct$treatmentVideo==1 & data_funct$treatmentLongSurvey==1,]

  vecSEs=foreach(i=1:S_funct, .combine='cbind', .export='estimateSE') %dopar% {
    set.seed(i) #Set seed here for reproductibility
    print(i)

    #Draw data with replacement
    bootdata_SHORT_NOVID_funct=data_SHORT_NOVID_funct[sample(nrow(data_SHORT_NOVID_funct), N_funct, replace=TRUE), ]
    bootdata_SHORT_VID_funct=data_SHORT_VID_funct[sample(nrow(data_SHORT_VID_funct), N_funct, replace=TRUE), ]
    bootdata_LONG_VID_funct=data_LONG_VID_funct[sample(nrow(data_LONG_VID_funct), N_funct, replace=TRUE), ]
    databoot_funct=rbind(bootdata_SHORT_NOVID_funct,bootdata_SHORT_VID_funct,bootdata_LONG_VID_funct)

    estimateSE(B_funct=B_funct,data_funct=databoot_funct)

  }

  return(vecSEs)
}

StoreVecRes=matrix(nrow=S,ncol=length(N_vec),data=NA)

#Run the precision estimation
cores=detectCores() #Number of cores
cl <- makeCluster(cores[1]-2, setup_timeout = 0.5)
registerDoParallel(cl)
matResults=matrix(data=NA,ncol=4,nrow=length(N_vec)) #To store the results
rownames(matResults)=N_vec
colnames(matResults)=c("N","Average SE","SE 2","SE 3")
matResults[,1]=N_vec
loop_counter=1
for(N_loop in N_vec){
  resVecLoop=estimatePrecisionParallel(N_funct=N_loop,S_funct=S,B_funct=B, data_funct=mydata)
  matResults[loop_counter,2]=mean(resVecLoop,rm.na=TRUE)
  matResults[loop_counter,3]=mean(ifelse(resVecLoop<=0.02,1,0),rm.na=TRUE)
  matResults[loop_counter,4]=mean(ifelse(resVecLoop<=0.03,1,0),rm.na=TRUE)
  StoreVecRes[,loop_counter]=resVecLoop
  loop_counter=loop_counter+1
}
stopCluster(cl)

#Show results
matResults

#Minimal SE L* where 80% SE<=L*
L_max=1000
minimalSEMat=matrix(data=NA,nrow=L_max,ncol=length(N_vec)+1)
colnames(minimalSEMat)=c("MaxSE",N_vec)
minimalSEMat[,1]=seq(1:L_max)/10000
for(k in 1:dim(minimalSEMat)[1]){
```

```
  for(j in 1:dim(StoreVecRes)[2]){
    minimalSEMat[k,j+1]=mean(ifelse(StoreVecRes[,j]<=minimalSEMat[k,1],1,0),na.rm
   = TRUE)
  }
}
minimalSEMat

minimalSEMat[122,] #Effect size of 2 percentage points
minimalSEMat[183,] #Effect size of 3pp
minimalSEMat[244,] #Effect size of 4pp
minimalSEMat[305,] #Effect size of 5pp

#Results
resultsLstar=matrix(data=NA,nrow=length(N_vec),ncol=3)
rownames(resultsLstar)=N_vec
colnames(resultsLstar)=c("N_vec","Minimal SE 80%","Minimal SE 95%")
resultsLstar[,1]=N_vec
for(k in 1:length(N_vec)){
  resultsLstar[k,2]=minimalSEMat[min(which(minimalSEMat[,k+1] > 0.80)),1]
  resultsLstar[k,3]=minimalSEMat[min(which(minimalSEMat[,k+1] > 0.95)),1]
}
resultsLstar
```

Listing 1: Ex-ante power analysis

Supplementary Materials 4: Filler questionnaire - Transportation choices

Questionnaire about transportation mode choices

Assume that you can travel privately and at your own expense. You are traveling alone from a point of departure A to a destination B. To make this journey, you have two modes of transport available to you: air and rail.

[Version T1, T3] These modes of transport are associated with two characteristics of the journey: the duration (indicated as door-to-door time) and the cost (integrating the cost of the connections between the place of departure, the station/airport and the station/airport-place of arrival).

[T2 and T4 versions] These modes of transport are associated with three characteristics of the journey: the duration (indicated as door-to-door time), the cost (integrating the cost of the connections between the place of departure/station/airport and the station/airport/place of arrival) and information on the proportion of people who choose the plane and the train.

The options are numerous, so they will be presented to you two by two.

You will have 10 choices to make. For each of them, you are invited to choose, among the 2 options, the one you prefer. There is no right or wrong answer, only your personal opinion. Please note that once a choice has been validated, it is no longer possible to go back and change it.

[Addition for T3, T4] For your information: CO_2 is a greenhouse gas emitted by vehicles used in transportation. The high level of greenhouse gas emissions in the atmosphere (such as CO_2) is the cause of dangerous climate changes for the planet. Many consequences have already been observed by climatologists, such as the melting of glaciers, the rise in water levels, or the increase in frequency and intensity of extreme weather events (fires, floods...).

We observe that currently, for the same distance, the plane is about 20 times more polluting per passenger than the train.

Trip characteristics

Mode: long-distance mode of transport used for this trip

Duration: total travel time in hours, **including connection time from the departure point to the station/airport and station/airport to the arrival point, as well as waiting and boarding times.**

Cost: total cost in euros of the trip, **including the cost of the connection between home and train station/airport and train station/airport and home.**

[T2 and T4] **Share of passengers choosing the option:** information given as an indication of the percentage (rounded to the nearest 10%) of passengers choosing each of the two options for the trip considered.

Here is an example

[T1, T3 and T5 versions]

	Option 1	Option 2
**Mode**	Plane	Train
**Duration***	2h10	2h50
**Cost****	100 €	50 €

* Door-to-door time

** Total cost including the cost of connections

Choosing option 1 means that you prefer the plane trip, which takes 2h10 and costs 100€.

Choosing option 2 means that you prefer the train trip, which takes 2h50 and costs 50€.

[T2 and T4 versions]

	Option 1	Option 2
**Mode**	Plane	Train
**Duration***	2h10	2h50
**Cost****	100 €	50 €
**Proportion of people choosing the option**	10%	90%

* Door-to-door time

** Total cost including the cost of connections

Choosing option 1 means that you prefer the plane trip, which takes 2h10, costs 100€. The proportion of people who choose the plane for this trip is 10%.

Choosing option 2 means that you prefer to travel by train, which takes 2h50 and costs 50€. The proportion of people who choose the train for this trip is 90%.

[For all choices, block backspace].

[To insert: 10 CHOICES (non-random order of the cards)]

[T1 and T3 versions] Here is the last choice you were offered:

[For block 1 respondents]

	**Option 1**	**Option 2**
**Mode**	Plane	Train
**Duration***	3h20	4h10
**Cost****	75 €	250 €

[For block 2 respondents]

	**Option 1**	**Option 2**
**Mode**	Plane	Train
**Duration***	2h10	4h10
**Cost****	75 €	250 €

For this trip, what would be according to you the proportion of people (on a representative sample of the French population) to choose the plane compared to the train for this alternative (to the nearest 10%)?

[In the form of a check box (single answer) or in the form of a cursor].

Distribution of people between plane and train	0% plane 100% train	10% plane 90% train	20% plane 80% train	30% plane 70% train	40% plane 60% train	50% plane 50% train	60% plane 40% train	70% plane 30% train	80% plane 20% train	90% plane 10% train	100% plane 0% train
Your estimate											

Which means of transportation do you use for your **private trips** (leisure, family, etc.), **lasting at least 2 hours** (door-to-door)? For each of the proposed modes, please indicate how often you use them for your private trips.

	Never	1 to 3 times every 5 years	Once per year	3 to 6 times per year	Once per month	2 to 3 times per month	Once per week or more	More than 3 times per week
Car								
Train								
Plane								

You will now answer a series of questions in the form of lists of propositions for which you will have to give your opinion. There are no right or wrong answers, only your opinion. Choose the answers that are the closest to your opinion.

	Strongly disagree	Somewhat disagree	Neither agree nor disagree	Somewhat agree	Strongly agree
Taking the plane is pleasant					
Taking the train is pleasant					
Taking the plane is seen positively by my family and friends					
Taking the train is seen in a positive light by my family and friends					
Thinking about the plane evokes positive emotions					
Thinking about the train evokes positive emotions					
My family and friends fly regularly					
My relatives take the train regularly					
My family and friends have a positive view of flying					
My family and friends have a positive view of taking the train					

To what extent would you say you are concerned about the impact of your travel on climate change? Check the box next to the statement that best describes your opinion.

☐ Not at all concerned
☐ Somewhat unconcerned
☐ Neither concerned nor not concerned
☐ Somewhat concerned
☐ Very concerned

Do you have relatives abroad?

☐ Yes
☐ No

[If yes to the previous question]: Are these relatives located in countries not accessible by train or car?

☐ Yes
☐ No

Supplementary Materials 5: Prediction survey

------SCREEN 1-----

Hello,

Thank you very much for agreeing to participate in this survey conducted by French academics. In the following screens, we will ask you several questions about the impact of L214 videos on the French population. More precisely, the objective for you will be to predict the impact of a video showing animals' mistreatment in intensive farms on the reactions of French people. We ask you to answer as accurately as possible and to try to predict the impact of this type of video.

For those who will have answered this survey before **January 31st**: we will randomly select a participant and we will randomly select one of his predictions. If this prediction is close enough to reality (i.e. within 5 percentage points of the correct answer), we will donate 100€ to the L214 association.

The results of this study will be communicated to you once the study is completed.

The study should last about 10 minutes.

The information provided in this questionnaire will only be used for academic/scientific purposes (no commercial purposes).

Confidentiality, Disclosure of Information, and Voluntary Participation Statement
For the purpose of this survey, we will use the email address you provided when you registered to retrieve your responses to the registration form. Once the data is retrieved, your email address will be deleted from the database. The anonymized data will then be indefinitely stored on a data sharing account for replication purposes and to promote reproducible science. Participation is entirely voluntary. You may end your participation at any time.

If you have any questions about this study, you can contact Sylvie Borau: s.borau@tbs-education.fr

Purpose of processing: The information collected about you will be processed for the research team led by Sylvie Borau (Toulouse Business School, 20 Bd Lascrosses, 31000 Toulouse, France; CIRED, 45bis Av. de la Belle Gabrielle, 94130 Nogent-sur-Marne)

The CNRS Data Protection Officer can be contacted for any questions on the protection of personal data. Its contact details are as follows: Service protection des données, 2 rue Jean Zay, 54519 Vandoeuvre-lès-Nancy ; mail : dpd.demandes@cnrs.fr.

Personal data is collected and processed for the following purpose: an exploration of animal activism strategies.
Recipients of the data: Depending on their respective needs, the recipients of all or part of the data are: the research team led by Sylvie Borau. No transfer of data outside the European Union is carried out. Your personal data will be kept for a period of: 36 months (duration of the research project).
You have the following rights for the use made of your data:
- The right of opposition: you can at any time oppose the processing of your data and have the right to withdraw your consent (depending on the case)
- The right to access and rectify your data
- The right to erasure
- The right to restricted use when your data is not necessary or no longer useful –
You can exercise these rights by contacting Sylvie Borau: s.borau@tbs-education.fr

You can also contact your DPO at the following address: CNRS Data protection department – 2 rue Jean Zay – 54519 – Vandoeuvre lès Nancy - dpd.demandes@cnrs.fr
If you believe, after contacting us, that your computer rights and freedoms are not respected, you have the possibility of lodging a complaint online with the CNIL or by post.

Before you start answering this questionnaire, could you please fill in your email address in order to receive the results of this study and validate your pre-registration for this study.

/____/

------ SCREEN 2-----

Risks: In the following questionnaire, you will view an excerpt from an L214 video that contains explicit images that may arouse unpleasant levels of emotion. These feelings are generally temporary.

○ I am aware of the explicit content of this study

Consent: I have read this form and am aware that I am being asked to participate in a study that will contain explicit images. I voluntarily agree to participate in this study. I may end my participation at any time.

○ I agree to participate in this study

○ I do NOT agree to participate in this study

----- SCREEN 3----

As a last step before starting the study, we would like to make sure that you can listen to sounds on your device.

Below, you will be asked to listen to a sound and then indicate the sound you heard.

Sound test: Please listen to this sound clip and indicate which sound it is by selecting the correct answer below.

○ Audible car alarm

○ Crickets

○ A meowing cat

○ A person who whistles

○ A barking dog

----- SCREEN 4----

Design of the study to which participants responded.
Please read the following information about the study design carefully. Then, you will have to answer a short comprehension test to check your knowledge.

The objective of our research is to understand the impact of L214 videos on public opinion. To do so, we created an online experiment in which a representative sample of the French population participated between Dec 4th and Dec 18th (3,300 respondents in total).

In our experiment, we randomly assigned participants to three groups.

1) In the first group, participants were not exposed to any video. We just asked them to report their emotional state. Then we presented them with a list of animal charities and petitions to which they could donate money.
2) In the second group, participants began by watching an excerpt from an L214 video. We then asked them the same questions as in the first group about their emotional state and about donations.
3) In the third group, the participants also started by watching the L214 video clip, but they then answered an 3 ~ 4 minute questionnaire on a topic unrelated to the video (they had to choose between taking a plane or a train for fictional trips). Only after having answered this questionnaire on the modes of transportation, we asked them the same questions as in the first two groups on their emotional state and on donations.

To summarize, respondents were randomly assigned to one of three groups:

Recap table

	**Group 1**	**Group 2**	**Group 3**
**Video L214** 📺	X	Vidéo L214	Vidéo L214
**Transportation survey** 🚄✈️	X	X	Questionnaire of 3 ~ 4 mn related to transportation
**Emotional state** ❤️	Questions relatives à leur état émotionnel	Questions relatives à leur état émotionnel	Questions relatives à leur état émotionnel
**Donations for animals** €	Donations to associations and petitions	Donations to associations and petitions	Donations to associations and petitions

----- SCREEN 5----

Understanding of the study design

Please answer the following question to demonstrate your understanding of the design of this study:

Which of the following statements are true? Please check all that apply.

◯ In all 3 groups, participants were exposed to a video from L214 before answering the questionnaire.

◯ In all 3 groups, participants were asked to answer questions about their emotional state.

◯ In all 3 groups, we presented a list of animal charities and petitions for which respondents could donate money.

◯ In all 3 groups, participants were asked to complete a questionnaire about transportation.

◯ In all 3 groups, respondents were asked to predict the emotional state of French people exposed to a video from L214.

----- SCREEN 6----

Understanding the study design (2nd chance)

It seems that you did not answer perfectly correctly. Here is the summary table of the study design as a reminder.

Recap table

	**Group 1**	**Group 2**	**Group 3**
**Video L214** 📺	X	Vidéo L214	Vidéo L214
**Transportation survey** 🚄✈️	X	X	Questionnaire of 3 ~ 4 mn related to transportation
**Emotional state** ❤️	Questions relatives à leur état émotionnel	Questions relatives à leur état émotionnel	Questions relatives à leur état émotionnel
**Donations for animals** €	Donations to associations and petitions	Donations to associations and petitions	Donations to associations and petitions

Which of the following statements are true? Please check all that apply.

◯ In all 3 groups, participants were exposed to a video from L214 before answering the questionnaire.

◯ In all 3 groups, participants were asked to answer questions about their emotional state.

◯ In all 3 groups, we presented a list of animal charities and petitions for which respondents could donate money.

◯ In all 3 groups, participants were asked to complete a questionnaire about transportation.

◯ In all 3 groups, respondents were asked to predict the emotional state of French people exposed to a video from L214.

----- SCREEN 7----

Answers

Here are the responses regarding the study design:

◯ In all 3 groups, the participants were exposed to a video of L214 before answering the questionnaire. -> **False**, only two groups were exposed to the L214 video before answering the questionnaire, one group was not exposed to any video.

◯ In all 3 groups, the participants had to answer questions related to their emotional state -> **True**

◯ In all 3 groups, we presented a list of animal charities and petitions for which respondents could donate money -> **True**

◯ In all 3 groups, participants were asked to complete a transportation questionnaire - > **False,** only one group completed a transportation questionnaire before answering questions about emotions and donations

◯ In all 3 groups, the respondents had to predict the emotional state of the French exposed to a video of L214 - > **False,** no group had to make predictions! On the other hand, this is what we ask you to do in this questionnaire. It's your turn now!

----- SCREEN 8----

Purpose of the survey

In what follows, we will ask you to predict as best as you can and as truthfully as you can the behavior of the participants in each of the three groups.

○ I understand the purpose of the survey.

----- SCREEN 9----

On the following page, you will discover the extract of the L214 video that the participants of the 2nd and 3rd groups watched.

The investigation of the L214 association took place over several months and used hidden cameras.

Please watch this video carefully. Please note that the video is only one minute and 20 seconds long.

----- SCREEN 10----

VIDEO

----- SCREEN 11----

First, we ask you to predict the responses of the participants in the **1st group (reminder: these participants have not seen the video).**

Regarding the emotional state, participants were asked to indicate the extent to which they felt the following emotions - with images of faces experiencing these emotions: anger, disgust, fear, sadness, surprise, and happiness. From the data collected, we created a negative emotional state indicator that is equal to the sum of the scores assigned to the negative emotions (anger, disgust, fear, sadness, surprise) minus the score assigned to happiness. **This emotion score that we have constructed takes values between 0% (very negative emotional state) and 100% (very positive emotional state).**

According to you, what is the average emotion score of the participants in the 1st group (no video)?
This emotion score takes values between 0% (very negative emotional state) and 100% (very positive emotional state). A higher score reflects a stronger positive emotion.

How confident are you in your answer? Please indicate your level of confidence on a scale from1 = "Not at all confident" to 10 = "Extremely confident".

1	2	3	4	5	6	7	8	9	10
“**Not at all confident**”									" **Extremely confident** "

As for donations, participants in the experiment were given 50 cents. They could decide to keep this money (and cash it at the end of the experiment) or to donate part of it. We presented 7 donation options to the participants (4 associations and 3 petitions). The donations for the petitions were intended to increase their visibility on social networks. Participants were informed that only one of their decisions would be implemented at the end of the study but they did not know which association or petition would be chosen. Participants were asked to indicate for each association/petition how much they would like to donate if that association or petition was chosen. Participants were informed that the selected association or petition had been chosen before the experiment. From the data collected, we created a donation indicator that represents the average percentage of a participant's donation. **This donation score takes values between 0% (out of the 7 associations/petitions, the participant did not donate anything to any of the associations/petitions) and 100% (out of the 7 associations/petitions, the participant systematically donated all his money, i.e. 50 cents). A score of 40% means that the participant has given on average 40% of his money on the 7 associations/petitions considered.**

List of proposed French associations:

- Assiettes Végétales: An association that seeks to put more vegetarian and vegan meals in university canteens.
- L214: An association for animal protection which denounces the cruelest practices of the breeding industry and seeks to develop food without animal products
- SPA: An association that works for the protection of animals, mainly domestic animals (cats, dogs), by fighting for example against abandonment and by promoting adoptions.
- Welfarm: An association that supports the initiatives of breeders and the agri-food industry that contribute to improving the well-being of animals in breeding

List of petitions:

- Against intensive breeding: A petition to ban intensive livestock farming in France
- For vegetarian meals: A petition to have at least one vegetarian meal per day in all public canteens in France
- To protect activists: A petition to protect activists in France who record videos showing the mistreatment of animals in intensive farming.

According to you, what is the average donation score of the participants in the 1st group (no video)?

This donation score takes values between 0% (out of the 7 associations/petitions, the participant did not donate anything to any of the associations/petitions) and 100% (out of the 7 associations/petitions, the participant systematically donated all his money). A score of 40% means that the participant gave an average of 40% of his money on the 7 associations/petitions considered. A higher score reflects the fact that the participant gave more to the associations/petitions on average.

How confident are you in your answer? Please indicate your level of confidence on a scale from 1 = "Not at all confident" to 10 = "Extremely confident".

1	2	3	4	5	6	7	8	9	10
**“Not at all confident”**									**"Extremely confident"**

----- SCREEN 12----

We now ask you to predict the responses of **participants in the 2nd group (recall: these participants saw the video but did not answer the survey about transportation).**

What do you think is the average emotion score of the participants in the 2nd group after watching the video?
This emotion score takes values between 0% (very negative emotional state) and 100% (very positive emotional state). A higher score thus reflects a stronger positive emotion.

How confident are you in your answer? Please indicate your level of confidence on a scale from 1 = "Not at all confident" to 10 = "Extremely confident".

1	2	3	4	5	6	7	8	9	10
**"Not at all confident"**									**"Extremely confident"**

According to you, what is the average donation score of the participants in the 2nd group after having seen the video?

This donation score takes values between 0% (on the 7 associations/petitions, the participant did not donate anything to any of the associations/petitions) and 100% (on the 7 associations/petitions, the participant systematically donated all his money). A score of 40% means that the participant gave an average of 40% of his money on the 7 associations/petitions considered. A higher score reflects the fact that the participant gave more to the associations/petitions on average.

How confident are you in your answer? Please indicate your level of confidence on a scale from 1 = "Not at all confident" to 10 = "Extremely confident".

1	2	3	4	5	6	7	8	9	10
**"Not at all confident"**									**"Extremely confident"**

----- SCREEN 13----

We now ask you to predict the responses of the **participants in the 3rd group. As a reminder: these participants watched the video and then completed the transportation questionnaire before indicating their emotional state and donation choice**. On average, participants took 3 ~ 4 minutes to complete the transportation questionnaire. Only then did they report their emotional state and desired donations.

What do you think is the average emotion score of the participants in the 3rd group after watching the video and taking the transportation questionnaire?
This emotion score takes values between 0% (very negative emotional state) and 100% (very positive emotional state). A higher score thus reflects a stronger positive emotion.

How confident are you in your answer? Please indicate your level of confidence on a scale from 1 = "Not at all confident" to 10 = "Extremely confident"

1	2	3	4	5	6	7	8	9	10
“**Not at all confident”**									“**Extremely confident**”

In your opinion, what is the average donation score of the participants in the 3rd group after having seen the video and after having answered the questionnaire on transportation?
This donation score takes values between 0% (out of the 7 associations/petitions, the participant did not donate anything to any of the associations/petitions) and 100% (out of the 7 associations/petitions, the participant systematically donated all his money). A score of 40% means that the participant gave an average of 40% of his money on the 7 associations/petitions considered. A higher score reflects the fact that the participant gave more to the associations and petitions on average.

How confident are you in your answer? Please indicate your level of confidence on a scale from 1 = "Not at all confident" to 10 = "Extremely confident".

1	2	3	4	5	6	7	8	9	10
“**Not at all confident”**									“**Extremely confident**”

----- SCREEN 14----

Thank you for your participation in this study.

For any questions, you can contact Sylvie Borau: s.borau@tbs-education.fr
